# Leading trait dimensions in flood-tolerant plants

**DOI:** 10.1093/aob/mcac031

**Published:** 2022-03-08

**Authors:** Yingji Pan, Ellen Cieraad, Jean Armstrong, William Armstrong, Beverley R Clarkson, Ole Pedersen, Eric J W Visser, Laurentius A C J Voesenek, Peter M van Bodegom

**Affiliations:** Institute of Environmental Sciences (CML), Leiden University, Leiden, The Netherlands; Key Laboratory of Bio-Resource and Eco-Environment of Ministry of Education, College of Life Sciences, Sichuan University, Chengdu, China; Institute of Environmental Sciences (CML), Leiden University, Leiden, The Netherlands; Nelson Marlborough Institute of Technology, Nelson, New Zealand; Department of Biological Sciences, University of Hull, Hull, UK; School of Agriculture and Environment, The University of Western Australia, Perth, Australia; Department of Biological Sciences, University of Hull, Hull, UK; School of Agriculture and Environment, The University of Western Australia, Perth, Australia; Manaaki Whenua-Landcare Research, Hamilton, New Zealand; School of Agriculture and Environment, The University of Western Australia, Perth, Australia; Freshwater Biological Laboratory, University of Copenhagen, Copenhagen, Denmark; Experimental Plant Ecology, Institute for Water and Wetland Research, Radboud University, Nijmegen, The Netherlands; Institute of Environmental Biology, Utrecht University, Utrecht, The Netherlands; Institute of Environmental Sciences (CML), Leiden University, Leiden, The Netherlands

**Keywords:** Adaptations to stressful environments, flooding-induced traits, key trait dimensions, leaf economics traits, plant strategies and functioning, trait-based approaches

## Abstract

**Background and Aims:**

While trait-based approaches have provided critical insights into general plant functioning, we lack a comprehensive quantitative view on plant strategies in flooded conditions. Plants adapted to flooded conditions have specific traits (e.g. root porosity, low root/shoot ratio and shoot elongation) to cope with the environmental stressors including anoxic sediments, and the subsequent presence of phytotoxic compounds. In flooded habitats, plants also respond to potential nutrient and light limitations, e.g. through the expression of leaf economics traits and size-related traits, respectively. However, we do not know whether and how these trait dimensions are connected.

**Methods:**

Based on a trait dataset compiled on 131 plant species from 141 studies in flooded habitats, we quantitatively analysed how flooding-induced traits are positioned in relation to the other two dominant trait dimensions: leaf economics traits and size-related traits. We evaluated how these key trait components are expressed along wetness gradients, across habitat types and among plant life forms.

**Key Results:**

We found that flooding-induced traits constitute a trait dimension independent from leaf economics traits and size-related traits, indicating that there is no generic trade-off associated with flooding adaptations. Moreover, individual flooding-induced traits themselves are to a large extent decoupled from each other. These results suggest that adaptation to stressful environments, such as flooding, can be stressor specific without generic adverse effects on plant functioning (e.g. causing trade-offs on leaf economics traits).

**Conclusions:**

The trait expression across multiple dimensions promotes plant adaptations and coexistence across multifaceted flooded environments. The decoupled trait dimensions, as related to different environmental drivers, also explain why ecosystem functioning (including, for example, methane emissions) are species and habitat specific. Thus, our results provide a backbone for applying trait-based approaches in wetland ecology by considering flooding-induced traits as an independent trait dimension.

## INTRODUCTION

By definition, flooding encompasses the hydrological conditions of waterlogging, partial or complete submergence (Sasidharan *et al.*, 2017). Freshwater flooding induces physical stress in plants, but additionally induces anoxic soil conditions. The distinct biogeochemical processes and their phytotoxic products associated with anaerobic metabolic pathways can also have adverse impacts on plant survival in flooded habitats (Greenway *et al.*, 2006; Voesenek *et al.*, 2006; Pezeshki and DeLaune, 2012). In response, some plant species have specific traits to cope with flooding-induced conditions, not only via facilitating oxygen transport to the anoxic rhizosphere, but also via ameliorating the phytotoxic compounds produced during and after flooding periods (Armstrong *et al.*, 1994; Visser *et al.*, 2000; Colmer and Voesenek, 2009; Voesenek and Bailey-Serres, 2013). Under the influence of the gaseous phytohormone ethylene, produced shortly after exposure to anoxic conditions, fast shoot elongation and adventitious root development are stimulated, which are costly for long-term adaptation (Blanch *et al.*, 1999; Voesenek *et al.*, 2004). These more permanent flooding-induced traits, including root porosity, changed root/shoot ratios and shoot elongation, have been intensively examined in eco-physiological studies (Voesenek and Bailey-Serres, 2015; Winkel *et al.*, 2016; Moor *et al.*, 2017). Root porosity reflects the proportion of longitudinally interconnected gas-filled spaces in root tissues enhancing gas diffusion, which improves plants’ performance in flooded conditions (Armstrong, 1980; Justin and Armstrong, 1987; Colmer, 2003*b*; Garssen *et al.*, 2015). Amongst others, the root/shoot ratio is shaped by environmental drivers such as light, nutrients and different types of ecosystems (Valladares *et al.*, 2000; Cakmak *et al.*, 2007). Plants tolerant to flooding tend to have a reduced root/shoot ratio to increase oxygen access (have more shoot biomass) and reduce oxygen sinks (have less root biomass) to tolerate flooding (Idestam-Almquist and Kautsky, 1995; Lopez and Kursar, 2003; Jung *et al.*, 2009). Therefore, the root/shoot ratio also reflects the oxygen balance between different plant organs in flooding-induced conditions (Mommer *et al.*, 2004; van Bodegom *et al.*, 2005; Stromberg and Merritt, 2016). Once submerged, shoot elongation is a trait helping flooding-tolerant plant shoot tips to quickly reach above the water surface to restore contact with the atmosphere (Voesenek *et al.*, 2003; Nagai *et al.*, 2010). While previous studies have assessed flooding-induced trait expression for a single or few species, the lack of integrative analyses forms a major barrier to the application of such elaborate observations at a broader scale (Moor *et al.*, 2017; Pan *et al.*, 2019).

In addition to dealing with flooding-induced stressors, plants may have to adapt to habitat resources mainly including nutrients and light. The leaf economics spectrum (Wright *et al.*, 2004) expresses traits [such as specific leaf area (SLA) and leaf nitrogen content] that allow us to distinguish plant strategies based on investment and turnover of resources to leaves, providing a spectrum from conservative to acquisitive strategies (Reich *et al.*, 1997; Wright *et al.*, 2004; Reich, 2014). Size-related traits (such as plant height and seed mass) are considered as another important but independent trait dimension expressing responses to competition for light and water (Diaz *et al.*, 2016). The quantitative analysis of these two trait dimensions helps us to understand the fundamental strategies for plant growth, survival and reproduction (e.g. van Bodegom *et al*., 2012). Applications of these two trait dimensions have led to increased insights into critical ecosystem processes, such as the feedbacks between litter decomposition and fire regimes (Cornelissen *et al.*, 2017). However, the pattern of dominant trait dimensions in plants adapted to flooding stress is unknown.

**Fig. 1.**
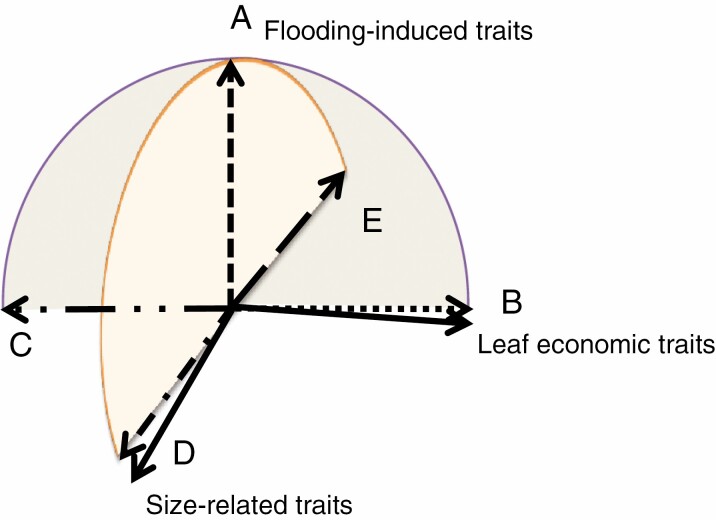
Possible positions of flooding-induced traits (dashed lines) relative to the leaf economics trait and size-related trait axes (solid lines). If adaptation to wetlands does not intrinsically hinder plant functions of resource acquisition or allocation, then flooding-induced traits should be decoupled from the leaf economics trait axis (A). If adaptation to wetlands facilitates plant functioning in terms of nutrient acquisition and allocation, then flooding-induced traits should be positively correlated to the leaf economics trait axis (B). If adaptation to wetlands is costly and causes trade-offs on leaf nutrient functioning, then flooding-induced traits should be negatively correlated to the leaf economics trait axis (C). If the choices of varied wetland-specific strategies are dependent on the plant size, then flooding-induced traits should be correlated to the size-related trait axis (D and E).

As flooding-induced traits play important but distinct roles compared with leaf economics traits and size-related traits in plant functioning, understanding whether and how these different groups of traits position relative to each other will improve our knowledge of plant strategies that cope with flooding stresses, in combination with differences in nutrient and light availability. If flooding-induced traits are decoupled from leaf economics traits, this suggests that flooding-induced traits are cheap to develop without trade-offs in nutrient acquisition or allocation (Fig. 1A). Plants would therefore not be constrained by habitat fertility when responding to flooding stress. On the other hand, if flooding-induced traits are positively co-ordinated to leaf economics traits, it indicates that adaptation to flooding stress facilitates the functioning of the leaf economics spectrum (Fig. 1B). Alternatively, if flooding-induced traits and leaf economics traits are negatively co-ordinated, it suggests that plants have to sacrifice part of their leaf resources as a cost of responding to flooded conditions (Fig. 1C). If flooding-induced traits are tightly co-ordinated with size-related traits, it suggests that either larger plants can easily outgrow the water column and profit more from aerenchyma tissues (Fig. 1D) or that plants that are more tolerant to flooding stress need less shoot elongation in order to reach out of the water surface for light and gases (Fig. 1E).

So far, some arguments are available that support the case of decoupled relationships (as shown in Fig. 1A). One line of reasoning is that the environmental drivers of the different trait groups are different. With nutrient and water availability driving leaf economics traits, and light availability steering size-related traits (Reich, 2014; Diaz *et al.*, 2016), while water regimes and the subsequent oxygen availability modify flooding-induced traits (Colmer and Voesenek, 2009), we expect to see each trait group varying independently in response to its specific drivers. The second line of reasoning is that flooded habitats across the globe cover a wide fertility range (e.g. from oligotrophic bogs to eutrophic floodplains), which suggests a prevalence of trait decoupling: if there were to be significant trade-offs between flooding-induced traits and leaf economics traits, we should find plants to be constrained only to fertile flooded habitats in order to acquire sufficient nutrient resources in compensation for the costs on flooding-specific adaptations (Pan *et al.*, 2019). Despite these coincidental lines of evidence, a quantification of these trait dimensions and the relationships among them currently does not yet exist.

In this study, we hypothesize that flooding-induced traits are decoupled from leaf economics traits and size-related traits, and thus constitute a separate trait dimension independent from the other dominant trait dimensions (as shown in Fig. 1A). We expect such decoupled trait dimension to occur consistently across environmental gradients of nutrient and water in the various wetland habitat types analysed (i.e. from infertile bog to fertile marsh). We also expect to observe the general existence of the leaf economics spectrum in flood-tolerant species, considering the widespread ecological principles for budgeting of plant resources in varied ecosystems. In addition, we hypothesize that flooding-induced traits should contribute to plant habitat affinities across a wetness gradient.

To test these hypotheses, we analysed the dominant trait dimensions of seven key plant traits that are ecologically important and of which quantitative records were available. We used root porosity, root/shoot ratio and shoot elongation as representative of more permanent trait responses to flooding (Colmer and Voesenek, 2009; Voesenek and Bailey-Serres, 2015); leaf nitrogen (leaf N), leaf phosphorus (leaf P) and SLA to represent leaf economics traits (Wright *et al.*, 2004; Pan *et al*., 2020a); and plant height as representative of size-related traits (Diaz *et al.*, 2016). Furthermore, we tested individual trait–trait relationships between the seven key plant traits in flood-tolerant plants and the role of individual flooding-induced traits in contributing to habitat affinities across a wetness gradient. We envisage that this study will inspire research on adaptation to environmental stresses in other ecosystems.

## MATERIALS AND METHODS

### Data compilation

We compiled functional traits on plants recorded in flooded habitats, following the definition of the international Ramsar Convention (Ramsar Convention Secretariat, 2013) and the terminology guidance on the definition of ‘flooding’ (Sasidharan *et al.*, 2017), for both field and laboratory measurements based on a combination of expert knowledge of the existing literature and systematic searches in the Web of Science and Google Scholar. The literature search included, but was not limited to, the following key words: wetland, marsh, bog, floodplain, macrophytes, aquatic plants, hydrophyte, submerged, floating-leaved, emergent, isoetid, mangrove, root porosity, root/shoot ratio, shoot elongation, leaf N, leaf P, SLA, leaf dry matter per unit area (LMA) and plant height. We also checked the references of several important reviews of eco-physiological traits for wetlands and flooding events in the last 15 years (e.g. Voesenek *et al.*, 2006; Bailey-Serres and Voesenek, 2008; Voesenek and Bailey-Serres, 2015). Moreover, we circulated enquiries around our network of wetland/aquatic plant experts for recommendations for literature that we had possibly overlooked. We used The Plant List to eliminate synonyms in species names from our database (http://www.theplantlist.org).

Root porosity was quantified mainly as either the percentage of the hollow area in the root cross-section or the ratio of hollow volume to the whole root volume. These two methods generally show agreement in air-filled root porosity (Van Noordwijk and Brouwer, 1988). Root/shoot ratio was defined as the root dry mass divided by the shoot dry mass. Shoot elongation was calculated as the percentage of the maximum shoot length increase after submergence/flooding. We are aware that there are various other flooding-induced traits (e.g. radial oxygen loss and leaf gas films) that have been emphasized in eco-physiological studies. However, they are either qualitative or are represented in our database by too few consistently measured observations to be included in our statistical analysis.

To evaluate potential drivers of trait–trait relationships, we included habitat wetness, habitat type and growth form in our analysis.

The Ellenberg moisture indicator values provide insights into the extent to which species are known to occur at different extents of habitat wetness (Ellenberg, 1988). These indicator values are based on expert knowledge of the generic distribution of plant species along a gradient of habitat wetness, categorized into 12 levels from very dry habitats (level 1) to strictly aquatic (level 12). To make the Ellenberg moisture indicator applicable for a global analysis, we related the Ellenberg moisture indicator values to the USDA wetland plant classification to derive Ellenberg values for the flora of the USA (see details in Supplementary data Appendix A).

To obtain more comprehensive insights into the relationships between the traits of the species and the ecological backgrounds relevant to wetland conditions, we also recorded the habitat type for each trait observation according to a modified Ramsar classification as presented in Pan *et al*. (2020b) and we added life form to each plant species based on the descriptions in the original literature.

**Fig. 2.**
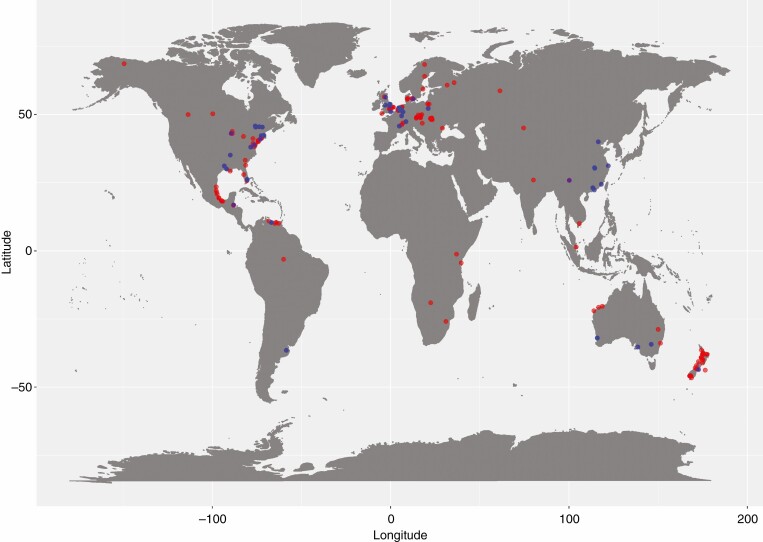
The location of the sampling sites. The field measurement data and laboratory measurement data are presented as red and blue dots, respectively. Note that the symbols are translucent and that brighter symbols indicate observations/studies at locations in close proximity to one another.

For this study, we took species mean trait values to allow analysis of trait–trait relationships, as individual studies did not provide all traits for the same situation (the distribution map of the sampling sites across the globe is shown in Fig. 2). Our analysis covered a total of 131 species of six life form categories (grass, sedge, emergent, submerged, floating-leaved and shrub/tree), with 113 species for root porosity, 60 species for root/shoot ratio and 32 species for shoot elongation (a list of the data sources and plant species can be found in Supplementary data Appendix B).

### Data analysis

To evaluate how flooding-induced traits relate to the other two trait dimensions at the interspecific level, we conducted a principal component analysis (PCA) in R (R Core Team, 2018). Due to gaps in the dataset, we could not run a PCA on all traits. Instead, we ran a PCA on each of the three flooding-induced traits separately with all leaf economics and size-related traits. Ellenberg moisture indicator values, habitat types and growth forms were used to label data points in the PCA to evaluate underlying patterns.

Then, we analysed individual trait–trait relationships between flooding-induced traits, leaf economics and size-related traits by standardized major axis (SMA) analysis (Warton *et al.*, 2006) to estimate how one trait scales against another (Warton *et al.*, 2012). The standardized axis slopes and coefficients of determination (*R*^2^) were calculated using the sma() function in the SMATR package (Warton *et al.*, 2012) in R (version 3.6.0) software (R Core Team, 2018). This analysis provides additional insights into the individual co-ordination, and also allows evaluation of the trait–trait relationships among flooding-induced traits and thus whether their relationship to leaf economics and size-related traits is consistent.

Finally, we ran an ordinary linear regression to examine how each flooding-induced trait relates to habitat wetness affinities (as represented by Ellenberg moisture indicator values). Data of the root/shoot ratio and shoot elongation were log_10_ transformed before analysis to comply with a normal distribution and homogeneity of variance. An alternative analysis, using a one-way analysis of variance (ANOVA) on groups of Ellenberg moisture indicator values, is presented in Supplementary data Appendix C, Table S1, and shows highly similar results.

## RESULTS

The PCA on each of the flooding-induced traits with the other two trait dimensions showed largely decoupled patterns. Leaf economics traits mainly occupied PCA axis 1, with size-related and flooding-induced traits on the other axes. The PCA loading scores on the first three PCA axes are shown in Table 1. The pattern suggests that adaptation to flooded conditions in general does not hinder plant functions in resource acquisition or allocation (the data points labelled with habitat type are provided in Supplementary data Appendix C, Fig. S1, and 3-D PCA plots can be found in Supplementary data Appendix C, Fig. S2).

**Table 1.** The loading scores of flooding-induced traits, leaf economics traits and size-related traits on the first three principal component analysis (PCA) axes

Root porosity				Root/shoot ratio				Shoot elongation			
	Axis 1	Axis 2	Axis 3		Axis 1	Axis 2	Axis 3		Axis 1	Axis 2	Axis 3
Root porosity	0.40	–0.42	–0.71	Root/shoot ratio	0.03	–0.80	–0.52	Shoot elongation	0.06	–0.87	0.14
Leaf N	–0.61	0.14	–0.09	Leaf N	0.58	0.26	–0.28	Leaf N	0.63	0.13	–0.16
Leaf P	–0.51	–0.21	–0.52	Leaf P	0.44	0.15	–0.50	Leaf P	0.50	–0.14	–0.67
SLA	–0.46	–0.31	0.07	SLA	0.57	0.00	0.35	SLA	0.40	0.38	0.47
Plant height	0.00	0.81	–0.46	Plant height	–0.37	0.52	–0.53	Plant height	–0.44	0.24	–0.54
Variation explained(%)	42.0	22.8	15.9	Variation explained (%)	33.4	20.6	19.6	Variation explained (%)	39.2	21.8	17.5

**Fig. 3.**
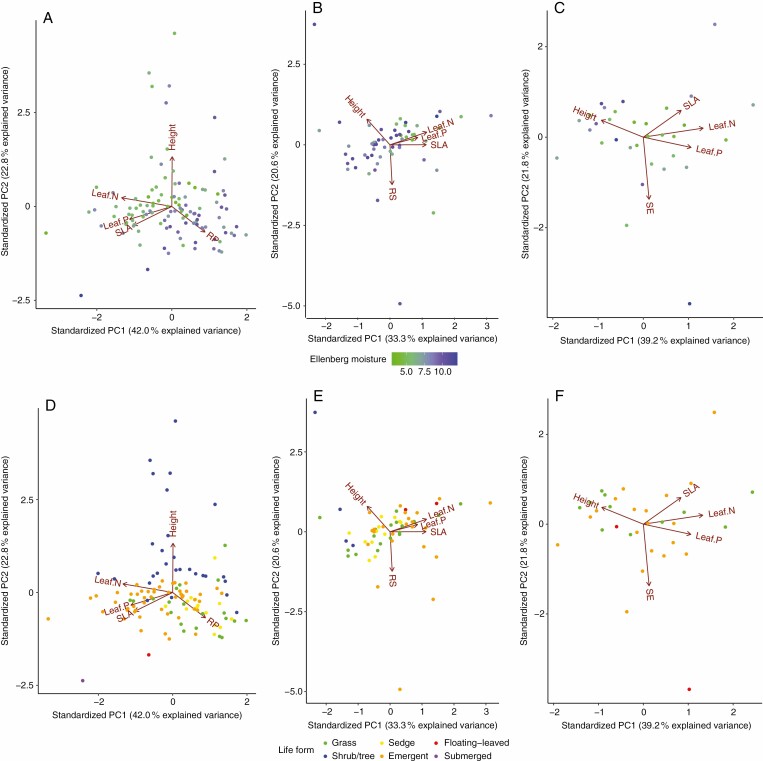
Principal component analysis (PCA) of leaf nitrogen (leaf N), leaf phosphorus (leaf P), specific leaf area (SLA), plant height (Height) and (A, D) root porosity (RP), (B, E) root/shoot ratio (RS) and (C, F) shoot elongation (SE). Each point represents one species, which is coloured according to its affinity for habitat wetness indicated by its Ellenberg moisture value (A–C) and life form (D–F), respectively. Supplementary data Appendix C, Fig. S1 presents figures with colours according to habitat type.

Root porosity was to a large extent decoupled from the leaf economics trait axis (as represented by leaf N, leaf P and SLA), but partly covaries with leaf N. The size-related trait (as represented by plant height) was positioned on a third trait axis. The first two PCA axes accounted for 42.0 and 22.8 % of the total variation, respectively (Fig. 3A). Also, the root/shoot ratio was to a large extent decoupled from the leaf economics trait axis and plant height as a size-related trait. The first two PCA axes accounted for 33.3 and 20.6 % of the total variation, respectively (Fig. 3B). A similar decoupled pattern applies to shoot elongation, except for some relationships with SLA. The first two PCA axes accounted for 39.2 and 21.8 % of the total variation, respectively (Fig. 3C). Root porosity tended to be lower in shrubs/trees than in grasses and sedges, while neither root/shoot ratio nor shoot elongation seemed strongly affected by life form (Figs. 3D–F) or environmental conditions as summarized by habitat type (Supplementary data Appendix C, Fig. S1).

The trait–trait relationships between flooding-induced traits, leaf economics traits and the size-related trait were further examined by the SMA analysis. The SMA analysis confirmed the largely decoupled nature of the three trait groups. Some exceptions included the significant relationships among three trait–trait pairs: root porosity–leaf N (*R*^2^ = 0.22, *P* < 0.05), root porosity–SLA (*R*^2^ = 0.09, *P *< 0.05) and SLA–plant height (*R*^2^ = 0.07, *P* < 0.05) (Table 2). In the case of root porosity–SLA and SLA–plant height, the explained variance was low.

**Table 2.** Trait–trait relationships

	Root porosity	Root/shoot ratio	Shoot elongation	SLA	Leaf N	Leaf P	Plant height
Root porosity		0.50 (0.37, 0.68)	1.15 (0.74, 1.80)	**–1.65 (–1.97, –1.38)**	**–2.74 (–3.23, –2.32)**	–1.86 (–2.23, –1.54)	–0.77 (–0.92, –0.63)
Root/shoot ratio	0.00 (*n* = 44)		–1.56 (–2.41, –1.01)	–1.68 (–2.17, –1.30)	–2.93 (–3.79, –2.27)	–1.94 (–2.51, –1.50)	–1.05 (–1.36, –0.81)
Shoot elongation	0.02 (*n* = 22)	0.02 (*n* = 23)		–2.04 (–2.90, –1.43)	–3.21 (–4.62, –2.23)	1.81 (1.26, 2.60)	–0.99 (–1.42, –0.69)
SLA	**0.09 (*n* = 113)**	0.03 (*n* = 60)	0.07 (*n* = 32)		**1.66 (1.43, 1.93)**	**1.07 (0.92, 1.26)**	**–0.47 (–0.56, –0.40)**
Leaf N	**0.22 (*n* = 113)**	0.03 (*n* = 60)	0 (*n* = 32)	**0.28 (*n* = 131)**		**0.65 (0.56, 0.75)**	–0.28 (–0.34, –0.24)
Leaf P	0.02 (*n* = 113)	0.02 (*n* = 60)	0.01 (*n* = 32)	**0.17 (*n* = 131)**	**0.25 (*n* = 131)**		0.44 (0.37, 0.52)
Plant height	0.00 (*n* = 113)	0.02 (*n* = 60)	0.02 (*n* = 32)	**0.07 (*n* = 131)**	0.00 (*n* = 131)	0.00 (*n* = 131)	

Trait–trait relaionships are shown between leaf economics traits: specific leaf area (SLA), leaf nitrogen (leaf N), leaf phosphorus (leaf P); flooding-induced traits: root porosity, root/shoot ratio, shoot elongation; and size-related trait: plant height. Traits were log_10_ transformed before analysis. The upper right section shows standardized major axis slopes with 95 % confidence intervals (referring to the *y* variable in the column and the *x* variable in the row). Coefficients of determination (*R*^2^) and sample sizes are given in the lower left section. Significant relationships (*P* < 0.01) are highlighted in bold.

The other flooding-induced traits (root/shoot ratio and shoot elongation) did not relate significantly to any leaf economics or size-related trait.

The SMA analysis confirmed the significant and strong relationships between the leaf economics traits (leaf N, leaf P and SLA) (Table 2). This suggests that the leaf economics spectrum is also maintained in flooding-tolerant plants.

In contrast, there was no significant relationship among any trait–trait pair of the flooding-induced traits tested (i.e. among root porosity, root/shoot ratio and shoot elongation; *P* > 0.05), and the *R*^2^ values of effect sizes were only 0.00–0.02 (Table 2).

**Fig. 4.**
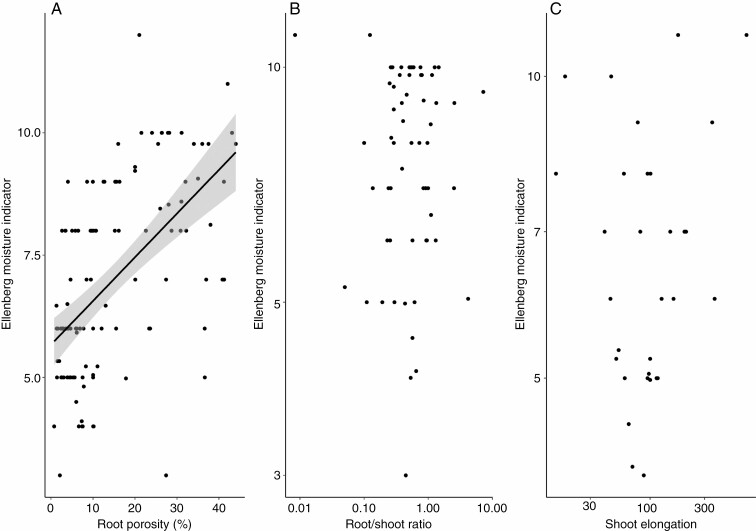
The linear relationships between habitat wetness affinities (represented by Ellenberg moisture indicator) and the three flooding-induced traits. For root porosity (*R*^2^ = 0.30, *P* < 0.001, *n* = 113), root/shoot ratio (*R*^2^ = 0.00, *P* = 0.98, *n* = 60) and shoot elongation (*R*^2^ = 0.00, *P* = 0.53, *n* = 32). The root/shoot ratio and shoot elongation are log-transformed before analysis to comply with a normal distribution and homogeneity of variance.

To understand how an individual flooding-induced trait contributes to the generic habitat affinities for wetness of plant species, we further tested the relationships between the Ellenberg moisture indicator and individual flooding-induced traits (Fig. 4). Among the three flooding-induced traits, root porosity showed a significant linear relationship with the Ellenberg moisture indicator with a reasonably high effect size (*R*^2^ = 0.30, *P* < 0.001). However, there was no relationship between the Ellenberg moisture indicator and root/shoot ratio (*R*^2^ = 0.00, *P* = 0.98), or between the Ellenberg moisture indicator and shoot elongation (*R*^2^ = 0.00, *P* = 0.53). Hence, among the three flooding-induced traits, variation in root porosity significantly contributed to habitat affinities of plant species along a wetness gradient. Even though the root/shoot ratio and shoot elongation are considered important flooding-induced traits, they were not directly related to the distribution of plants along a wetness gradient.

## Discussion

### Flooding-induced traits as a key strategy component in flooding-tolerant plant species

Our research reveals that the three important flooding-induced traits are in independent trait dimensions decoupled from the leaf economics and size-related trait dimensions (Fig. 3). Moreover, similar to the pattern in terrestrial systems (Diaz *et al.*, 2016), we found that leaf economics traits and size-related traits also remain largely decoupled from each other in flooded ecosystems. This pattern suggests that in addition to other dominant trait dimensions, flooding-induced traits play an important but different ecological role in adaptation to flooded conditions. As leaf economics traits are indicative of habitat fertility and corresponding nutrient resources, and size-related traits confer a competitive power for light (and water), flooding-induced traits mainly contribute to coping with flooded environments. Concurrently, this decoupling of flooding-induced traits from leaf economics traits may suggest that adaptations to flooded conditions are either inexpensive, or sufficiently beneficial to offset the costs of such adaptations on the plant overall resource budget. For example, the extra access to light, CO_2_ and O_2_ gained by shoot elongation may compensate the costs thereof (Colmer and Voesenek, 2009). The generally decoupled relationships between flooding-induced traits and leaf economics traits provide an explanation for the broad distribution of aquatic plants (Santamaría, 2002; Chambers *et al.*, 2008) as they allow plant species to occur across a range of flooding stressors and habitat nutrient limitations (e.g. from oligotrophic bogs to eutrophic floodplains). Moreover, considering the tight associations between leaf economics traits and the whole-plant economics spectra including root and stem traits (Freschet *et al.*, 2010), we speculate that flooding-induced traits may also be decoupled from these whole-plant traits if such spectra also exist in flooding-tolerant plants. Together, these results indicate that the flooding-tolerant strategies are a key dimension independent of other plant strategy components.

In nature, multiple environmental stressors, including drought, heat, freezing, shading, infertility and soil salinity, impose pronounced challenges to the adaptation and survival of plants (Bohnert *et al.*, 1995; Wolfe and Tonsor, 2014). While various adaptive mechanisms have been carefully examined from genetic, to morphological, to community points of view (Wolfe and Tonsor, 2014; Bechtold, 2018; Liu *et al.*, 2018), an integral perspective on a plant’s strategies as a whole is missing. From a trait-based perspective, the decoupled flooding-tolerant strategy in relation to other plant strategy components may have implications for traits specific to other stressful ecosystems, such as waxy leaves in deserts or dauciform roots under extreme phosphate deficiencies (e.g. Bakker *et al.*, 2005), as our research provides evidence that certain adaptive strategies to stressful habitats can be cheap without causing any trade-offs in plant general functioning. We hypothesize that such decoupled trait dimensions allow plants to adapt to multifarious niche dimensions and facilitate species coexistence in stressful habitats (Westoby *et al.*, 2002; Li *et al.*, 2015).

### Diverse plant strategies enable adaptations to a multifaceted stressful environment

Despite their similar functional roles in adapting to flooded conditions, the inter-relationships within the three flooding-induced traits were all non-significant and weak (Table 2, *P* > 0.05 with *R*^2^ ranges from 0.00 to 0.02). Moreover, while root porosity significantly contributes to the preference of plant species along a wetness gradient, root/shoot ratio and shoot elongation are not directly linked to the Ellenberg moisture indicator, life form or habitat type (Figs 3 and 4; Supplementary data Appendix C, Fig. S1). We provide three non-mutually exclusive explanations for the lack of correspondence between the root/shoot ratio and shoot elongation vs. habitat wetness: (1) the root/shoot ratio is known to be influenced by other stressors as well, such as light, nutrient and different types of ecosystems (Jackson *et al.*, 1996; Valladares *et al.*, 2000; Cakmak *et al.*, 2007); (2) all investigated traits are plastic, and mean species responses may not fully capture species responses to flooding-induced conditions; and (3) other (potentially less permanent) flooding-induced traits may be more strongly related to habitat wetness (but not yet available in global databases). However, even though flooding-induced traits are not all tightly aligned along a wetness gradient, these traits may still contribute in conjunction or accumulatively to adapting to the different stresses in a flooded environment.

While mostly decoupled, some links were observed between flooding-induced traits and specific leaf economics traits. For example, we detected a trade-off between root porosity and leaf N at the interspecific level (*R*^2^ = 0.22, *P* < 0.01; Table 2). The ecological causal links between root porosity and leaf N are complicated, and experimental evidence has often been contradictory. On the one hand, the formation of root porosity impedes the nutrient acquisition efficiency and will lead to trade-offs (Kirk, 2003; Hu *et al.*, 2014). On the other hand, the production of large numbers of laterals in response to flooded conditions may increase the root surface area for nutrient acquisition (Lissner *et al.*, 2003), which cause an indirect correlation between root porosity and leaf N. In addition, increased root porosity may enhance oxygen released from the root to oxidize NH_4_^+^ to NO_3_^–^. The produced nitrate is the main source of stable and storable N for plants (Kirk, 2003), and consequently improves leaf N levels. The balance between these pros and cons may differ depending on habitat, with different implications for the investment in root porosity formation and leaf N acquisition. Considering the even stronger correlations among leaf economics traits, i.e. leaf N–SLA (*R*^2^ = 0.28, *P *< 0.01) and leaf N–leaf P (*R*^2^ = 0.25, *P* < 0.01), and the extremely weak correlations of root porosity–leaf P (*R*^2^ = 0.02, *P* > 0.05) and root porosity–plant height (*R*^2^ = 0.00, *P* > 0.05), the trade-off between root porosity and leaf N does not fundamentally change our interpretation that root porosity is in a trait dimension decoupled from leaf economics traits (Fig. 3).

We also observed two significant but weak relationships between SLA and other trait groups, i.e. SLA–root porosity (*R*^2^ = 0.09, *P* < 0.01; Table 2) and SLA–plant height (*R*^2^ = 0.07, *P* < 0.01; Table 2). Even though the effect sizes are small (9 and 7 %, respectively), this highlights SLA as a trait inter-related with all three trait dimensions simultaneously. Previous studies indicate that the relationships between SLA and plant flooding tolerance can be either positive or negative depending on life form, season and community-weighted SLA (Huber *et al.*, 2009; Violle *et al.*, 2011; Douma *et al.*, 2012; Wright *et al.*, 2017). Even so, amphibious/aquatic plants in general have a higher SLA than terrestrial plants (Mommer and Visser, 2005; Pierce *et al.*, 2012; Purcell *et al.*, 2019). However, the relatively low effect size of the three trait–trait pairs does not fundamentally impair the overall decoupled pattern of the three trait dimensions.

In combination, the specific connections between different trait dimensions indicate that the adaptations to flooded conditions involve rather complex and multifarious strategies as expressed in different plant trait dimensions. Multiple trait dimensions contribute to ramified but accumulative functions to prosper in a flooded environment. We propose dedicated multitrait experiments to further examine these trait–trait relationships. Moreover, we advocate global initiatives to acquire a more comprehensive understanding of wetland/freshwater plant traits to investigate the complex processes of how environmental variables regulate plant trait expression under flooded conditions (Iversen *et al.*, 2021).

### Implications for ecosystem functioning

Clarifying the relationships between wetland-specific traits and leaf economics traits is also important for upscaling plant functional traits to wetland ecosystem processes, such as methane emissions (Pan *et al.*, 2019). For instance, the transport of oxygen to the rhizosphere by root porosity (Colmer, 2003*a*; Lai *et al.*, 2011) can suppress methane production processes that require strictly anoxic conditions. In contrast, leaf N and leaf P are indicative of organic matter quality to support decomposition processes (Hobbie, 2015) and may hence stimulate methane production by supplying nutrients to methane-producing archaea (van Bodegom and Scholten, 2001; Bhullar *et al*., 2013a). In addition, methane emissions may be further enhanced by the ‘chimney effect’ of wetland plants in facilitating methane transport to the atmosphere through formation of root and stem porosity (Bhullar *et al*., 2013a). These complex and contrasting driving factors make it difficult to quantitatively understand the facilitation vs. suppression effects of wetland plants on methane emissions (Bhullar *et al*., 2013a). The decoupled relationships between leaf economics traits and wetland-specific traits found in this study further add to the variation in the impacts of wetland plants on methane emissions. Our results thus highlight that both flooding-induced traits and other key traits need to be considered to adequately predict methane emissions (Sutton-Grier and Megonigal, 2011; Bhullar *et al*., 2013b).

### Conclusions

Our results reveal that flooding-induced traits are largely decoupled from leaf economics and size-related trait dimensions, which suggests that flooding-induced traits constitute a different plant trait dimension. This trait decoupling allows plant species to cope with the multifaceted stressful flooded environment (in terms of flooding, resources and competition). Our study indicates that no integral general strategy exists that perfectly explains the adaptation of plants to complex flooded environments. Instead, the multiple facets of flood tolerance plant strategies, as shown by the combination of functional traits including flooding-induced traits, leaf economics traits and size-related traits, together contribute to the survival of plants in complex flooded environments and help them prosper globally across a wide range of habitat fertilities. These insights provide a trait-based foundation towards understanding the general flood tolerance plant strategies and the functioning of flooded ecosystems, as well as adaptations to habitat stress in different ecosystems.

## SUPPLEMENTARY DATA

Supplementary data are available online at https://academic.oup.com/aob and consist of the following. Appendix A: deriving Ellenberg moisture indicator values for plant species in the analysis. Appendix B: a list of plant species name and traits analysed in this study. Appendix C: principal component analysis (PCA) of leading trait dimension and ANOVA.

mcac031_suppl_Supplementary_Appendix_S1

mcac031_suppl_Supplementary_Appendix_S2

mcac031_suppl_Supplementary_Appendix_S3

## Data Availability

The data can be accessed freely at Dryad data repository; https://doi.org/10.5061/dryad.51c59zw9v.

## References

[CIT0001] Armstrong W . 1980. Aeration in higher plants. Advances in Botanical Research 7: 225–332.

[CIT0002] Armstrong W , Brändle R, Jackson MB. 1994. Mechanisms of flood tolerance in plants. Acta Botanica Neerlandica 43: 307–358. doi:10.1111/j.1438-8677.1994.tb00756.x.

[CIT0003] Bailey-Serres J , Voesenek LACJ. 2008. Flooding stress: acclimations and genetic diversity. Annual Review of Plant Biology 59: 313–339. doi:10.1146/annurev.arplant.59.032607.092752. 18444902

[CIT0004] Bakker C , Rodenburg J, van Bodegom PM. 2005. Effects of Ca- and Fe-rich seepage on P availability and plant performance in calcareous dune soils. Plant and Soil 275: 111–122. doi:10.1007/s11104-005-0438-1.

[CIT0006] Bechtold U . 2018. Plant life in extreme environments: how do you improve drought tolerance? Frontiers in Plant Science 9: 1–8. 29868044 10.3389/fpls.2018.00543 PMC5962824

[CIT0007] Bhullar GS , Edwards PJ, Olde Venterink H. 2013 *a*. Variation in the plant-mediated methane transport and its importance for methane emission from intact wetland peat mesocosms. Journal of Plant Ecology 6: 298–304. doi:10.1093/jpe/rts045.

[CIT0008] Bhullar GS , Iravani M, Edwards PJ, Olde Venterink H. 2013 *b*. Methane transport and emissions from soil as affected by water table and vascular plants. BMC Ecology 13: 32. doi:10.1186/1472-6785-13-32. 24010540 PMC3847209

[CIT0009] Blanch SJ , Ganf GG, Walker KF. 1999. Growth and resource allocation in response to flooding in the emergent sedge *Bolboschoenus medianus*. Aquatic Botany 63: 145–160.

[CIT0010] van Bodegom PM , Scholten JCM. 2001. Microbial processes of CH_4_ production in a rice paddy soil: model and experimental validation. Geochimica et Cosmochimica Acta 65: 2055–2066.

[CIT0011] van Bodegom PM , de Kanter M, Bakker C, Aerts R. 2005. Radial oxygen loss, a plastic property of dune slack plant species. Plant and Soil 271: 351–364.

[CIT0500] van Bodegom PM , Douma JC, Witte JPM, Ordoñez JC, Bartholomeus RP, Aerts R. 2012. Going beyond limitations of plant functional types when predicting global ecosystem-atmosphere fluxes: Exploring the merits of traits-based approaches. Global Ecology and Biogeography 21: 625–636.

[CIT0012] Bohnert HJ , Nelson DE, Jensen RG. 1995. Adaptations to environmental stresses. The Plant Cell 7: 1099–1111. doi:10.1105/tpc.7.7.1099. 12242400 PMC160917

[CIT0013] Cakmak I , Hengeler C, Marschner H. 2007. Partitioning of shoot and root dry matter and carbohydrates in bean plants suffering from phosphorus, potassium and magnesium deficiency. Journal of Experimental Botany 45: 1245–1250.

[CIT0014] Chambers PA , Lacoul P, Murphy KJ, Thomaz SM. 2008. Global diversity of aquatic macrophytes in freshwater. Hydrobiologia 595: 9–26.

[CIT0015] Colmer TD . 2003 *a*. Long-distance transport of gases in plants: a perspective on internal aeration and radial oxygen loss from roots. Plant, Cell &Environment 26: 17–36.

[CIT0016] Colmer TD . 2003 *b*. Aerenchyma and an inducible barrier to radial oxygen loss facilitate root aeration in upland, paddy and deep-water rice (*Oryza sativa* L.). Annals of Botany 91: 301–309. doi:10.1093/aob/mcf114. 12509350 PMC4795684

[CIT0017] Colmer TD , Voesenek LACJ. 2009. Flooding tolerance: suites of plant traits in variable environments. Functional Plant Biology 36: 665–681. doi:10.1071/fp09144. 32688679

[CIT0018] Cornelissen JHC , Grootemaat S, Verheijen LM, et al 2017. Are litter decomposition and fire linked through plant species traits? New Phytologist 216: 653–669. 28892160 10.1111/nph.14766

[CIT0019] Diaz S , Kattge J, Cornelissen JH, et al 2016. The global spectrum of plant form and function. Nature 529: 167–171. doi:10.1038/nature16489. 26700811

[CIT0021] Douma JC , Bardin V, Bartholomeus RP, van Bodegom PM. 2012. Quantifying the functional responses of vegetation to drought and oxygen stress in temperate ecosystems. Functional Ecology 26: 1355–1365. doi:10.1111/j.1365-2435.2012.02054.x.

[CIT0022] Ellenberg HH . 1988. Vegetation ecology of central Europe. Cambridge: Cambridge University Press.

[CIT0024] Freschet GT , Cornelissen JHC, van Logtestijn RSP, Aerts R. 2010. Evidence of the ‘plant economics spectrum’ in a subarctic flora. Journal of Ecology 98: 362–373. doi:10.1111/j.1365-2745.2009.01615.x.

[CIT0025] Garssen AG , Baattrup-Pedersen A, Voesenek LACJ, Verhoeven JTA, Soons MB. 2015. Riparian plant community responses to increased flooding: a meta-analysis. Global Change Biology 21: 2881–2890. doi:10.1111/gcb.12921. 25752818

[CIT0026] Greenway H , Armstrong W, Colmer TD. 2006. Conditions leading to high CO_2_ (>5 kPa) in waterlogged–flooded soils and possible effects on root growth and metabolism. Annals of Botany 98: 9–32. doi:10.1093/aob/mcl076. 16644893 PMC3291891

[CIT0027] Hobbie SE . 2015. Plant species effects on nutrient cycling: revisiting litter feedbacks. Trends in Ecology and Evolution 30: 357–363. doi:10.1016/j.tree.2015.03.015. 25900044

[CIT0028] Hu B , Henry A, Brown KM, Lynch JP. 2014. Root cortical aerenchyma inhibits radial nutrient transport in maize (*Zea mays*). Annals of Botany 113: 181–189. doi:10.1093/aob/mct259. 24249807 PMC3864730

[CIT0029] Huber H , Jacobs E, Visser EJW. 2009. Variation in flooding-induced morphological traits in natural populations of white clover (*Trifolium repens*) and their effects on plant performance during soil flooding. Annals of Botany 103: 377–386. doi:10.1093/aob/mcn149. 18713824 PMC2707307

[CIT0030] Idestam-Almquist J , Kautsky L. 1995. Plastic responses in morphology of *Potamogeton pectinatus* L. to sediment and above-sediment conditions at two sites in the northern Baltic proper. Aquatic Botany 52: 205–216. doi:10.1016/0304-3770(95)00499-8.

[CIT0031] Iversen LL , Girón JG, Pan Y. 2021. Towards linking freshwater plants and ecosystems via functional biogeography. Aquatic Botany 176: 103454.

[CIT0032] Jackson RB , Canadell J, Ehleringer JR, Mooney HA, Sala OE, Schulze ED. 1996. A global analysis of root distributions for terrestrial biomes. Oecologia 108: 389–411. doi:10.1007/BF00333714. 28307854

[CIT0034] Jung V , Hoffmann L, Muller S. 2009. Ecophysiological responses of nine floodplain meadow species to changing hydrological conditions. Plant Ecology 201: 589–598.

[CIT0035] Justin SHFW , Armstrong W. 1987. The anatomical characteristics of roots and plant response to soil flooding. New Phytologist 106: 465–495. doi:10.1111/j.1469-8137.1987.tb00153.x.

[CIT0036] Kirk GJD . 2003. Rice root properties for internal aeration and efficient nutrient acquisition in submerged soil. New Phytologist 159: 185–194. doi:10.1046/j.1469-8137.2003.00793.x. 33873689

[CIT0038] Lai W-L , Wang S-Q, Peng C-L, Chen Z-H. 2011. Root features related to plant growth and nutrient removal of 35 wetland plants. Water Research 45: 3941–3950. doi:10.1016/j.watres.2011.05.002. 21640369

[CIT0041] Li L , McCormack ML, Ma C, et al 2015. Leaf economics and hydraulic traits are decoupled in five species-rich tropical–subtropical forests. Ecology Letters 18: 899–906. doi:10.1111/ele.12466. 26108338

[CIT0043] Lissner J , Mendelssohn IA, Lorenzen B, Brix H, McKee KL, Miao S. 2003. Interactive effects of redox intensity and phosphate availability on growth and nutrient relations of *Cladium jamaicense* (Cyperaceae). American Journal of Botany 90: 736–748. doi:10.3732/ajb.90.5.736. 21659170

[CIT0044] Liu R , Wang L, Tanveer M, Song J. 2018. Seed heteromorphism: an important adaptation of halophytes for habitat heterogeneity. Frontiers in Plant Science 871: 1–10. 10.3389/fpls.2018.01515 PMC6199896 30386364

[CIT0045] Lopez OR , Kursar TA. 2003. Does flood tolerance explain tree species distribution in tropical seasonally flooded habitats? Oecologia 136: 193–204. doi:10.1007/s00442-003-1259-7. 12743794

[CIT0047] Mommer L , Visser EJ. 2005. Underwater photosynthesis in flooded terrestrial plants: a matter of leaf plasticity. Annals of Botany 96: 581–589. doi:10.1093/aob/mci212. 16024559 PMC4247027

[CIT0048] Mommer L , Pedersen O, Visser EJW. 2004. Acclimation of a terrestrial plant to submergence facilitates gas exchange under water. Plant, Cell & Environment 27: 1281–1287. doi:10.1111/j.1365-3040.2004.01235.x.

[CIT0049] Moor H , Rydin H, Hylander K, Nilsson MB, Lindborg R, Norberg J. 2017. Towards a trait-based ecology of wetland vegetation. Journal of Ecology 105: 1623–1635. doi:10.1111/1365-2745.12734.

[CIT0050] Nagai K , Hattori Y, Ashikari M. 2010. Stunt or elongate? Two opposite strategies by which rice adapts to floods. Journal of Plant Research 123: 303–309. doi:10.1007/s10265-010-0332-7. 20354754

[CIT0051] Pan Y , Cieraad E, van Bodegom PM. 2019. Are ecophysiological adaptive traits decoupled from leaf economics traits in wetlands? Functional Ecology 33: 1202–1210.

[CIT0052] Pan Y , Cieraad E, Armstrong J, et al 2020 *a*. Global patterns of the leaf economics spectrum in wetlands. Nature Communications 11: 4519. doi:10.1038/s41467-020-18354-3. PMC7481225 32908150

[CIT0053] Pan Y , Cieraad E, Clarkson BR, et al 2020 *b*. Drivers of plant traits that allow survival in wetlands. Functional Ecology 34: 956–967. doi:10.1111/1365-2435.13541.

[CIT0054] Pezeshki SR , DeLaune RD. 2012. Soil oxidation–reduction in wetlands and its impact on plant functioning. Biology 1: 196–221. doi:10.3390/biology1020196. 24832223 PMC4009779

[CIT0055] Pierce S , Brusa G, Sartori M, Cerabolini BE. 2012. Combined use of leaf size and economics traits allows direct comparison of hydrophyte and terrestrial herbaceous adaptive strategies. Annals of Botany 109: 1047–1053. doi:10.1093/aob/mcs021. 22337079 PMC3310502

[CIT0056] Purcell AST , Lee WG, Tanentzap AJ, Laughlin DC. 2019. Fine root traits are correlated with flooding duration while aboveground traits are related to grazing in an Ephemeral Wetland. Wetlands 39: 291–302.

[CIT0057] Ramsar Convention Secretariat. 2013. The Ramsar Convention Manual: a guide to the Convention on Wetlands (Ramsar, Iran, 1971). Gland, Switzerland: RamsarConvention Secretariat.

[CIT0058] R Core Team. 2018. R: a language and environment for statistical computing. Vienna, Austria: R Foundation Statistical Computing. https://www.r-project.org/.

[CIT0059] Reich PB . 2014. The world-wide ‘fast–slow’ plant economics spectrum: a traits manifesto. Journal of Ecology 102: 275–301. doi:10.1111/1365-2745.12211.

[CIT0060] Reich PB , Walters MB, Ellsworth DS. 1997. From tropics to tundra: global convergence in plant functioning. Proceedings of the National Academy of Sciences, USA 94: 13730–13734. doi:10.1073/pnas.94.25.13730. PMC28374 9391094

[CIT0061] Santamaría L . 2002. Why are most aquatic plants widely distributed? Dispersal, clonal growth and small-scale heterogeneity in a stressful environment. Acta Oecologica 23: 137–154. doi:10.1016/s1146-609x(02)01146-3.

[CIT0062] Sasidharan R , Bailey-Serres J, Ashikari M, et al 2017. Community recommendations on terminology and procedures used in flooding and low oxygen stress research. New Phytologist 214: 1403–1407. 28277605 10.1111/nph.14519

[CIT0063] Stromberg JC , Merritt DM. 2016. Riparian plant guilds of ephemeral, intermittent and perennial rivers. Freshwater Biology 61: 1259–1275.

[CIT0064] Sutton-Grier AE , Megonigal JP. 2011. Plant species traits regulate methane production in freshwater wetland soils. Soil Biology and Biochemistry 43: 413–420. doi:10.1016/j.soilbio.2010.11.009.

[CIT0065] Valladares F , Wright SJ, Lasso E, Kitajima K, Pearcy RW. 2000. Plastic phenotypic response to light of 16 congeneric shrubs from a Panamanian rainforest. Ecology 81: 1925–1936. doi:10.1890/0012-9658(2000)081[1925:pprtlo]2.0.co;2.

[CIT0066] Van Noordwijk M , Brouwer G. 1988. Quantification of air-filled root porosity: a comparison of two methods. Plant and Soil 111: 255–258. doi:10.1007/bf02139949.

[CIT0067] Violle C , Bonis A, Plantegenest M, et al 2011. Plant functional traits capture species richness variations along a flooding gradient. Oikos 120: 389–398.

[CIT0068] Visser EJW , Colmer TD, Blom CWPM, Voesenek LACJ. 2000. Changes in growth, porosity, and radial oxygen loss from adventitious roots of selected mono- and dicotyledonous wetland species with contrasting types of aerenchyma. Plant, Cell & Environment 23: 1237–1245. doi:10.1046/j.1365-3040.2000.00628.x.

[CIT0069] Voesenek LACJ , Bailey-Serres J. 2013. Flooding tolerance: O_2_ sensing and survival strategies. Current Opinion in Plant Biology 16: 647–653. doi:10.1016/j.pbi.2013.06.008. 23830867

[CIT0070] Voesenek LACJ , Bailey-Serres J. 2015. Flood adaptive traits and processes: an overview. New Phytologist 206: 57–73. 25580769 10.1111/nph.13209

[CIT0071] Voesenek LACJ , Benschop JJ, Bou J, et al 2003. Interactions between plant hormones regulate submergence-induced shoot elongation in the flooding-tolerant dicot *Rumex palustris*. Annals of Botany 91: 205–211. 12509341 10.1093/aob/mcf116 PMC4244986

[CIT0072] Voesenek LACJ , Rijnders JHGM, Peeters AJM, van de Steeg HM, de Kroon H. 2004. Plant hormones regulate fast shoot elongation under water: from genes to communities. Ecology 85: 16–27.

[CIT0073] Voesenek LACJ , Colmer TD, Pierik R, Millenaar FF, Peeters AJM. 2006. How plants cope with complete submergence. New Phytologist 170: 213–226. 16608449 10.1111/j.1469-8137.2006.01692.x

[CIT0074] Warton DI , Duursma RA, Falster DS, Taskinen S. 2012. smatr 3 – an R package for estimation and inference about allometric lines. Methods in Ecology and Evolution 3: 257–259.

[CIT0075] Warton DI , Wright IJ, Falster DS, Westoby M. 2006. Bivariate line-fitting methods for allometry. Biological Reviews of the Cambridge Philosophical Society 81: 259–291. 16573844 10.1017/S1464793106007007

[CIT0076] Westoby M , Falster DS, Moles AT, Vesk PA, Wright IJ. 2002. Plant ecological strategies: some leading dimensions of variation between species. Annual Review of Ecology and Systematics 33: 125–159.

[CIT0077] Winkel A , Visser EJW, Colmer TD, et al 2016. Leaf gas films, underwater photosynthesis and plant species distributions in a flood gradient. Plant, Cell & Environment 39: 1537–1548. 10.1111/pce.12717 26846194

[CIT0078] Wolfe MD , Tonsor SJ. 2014. Adaptation to spring heat and drought in northeastern Spanish *Arabidopsis thaliana*. New Phytologist 201: 323–334. 24117851 10.1111/nph.12485

[CIT0079] Wright AJ , de Kroon H, Visser EJW, et al 2017. Plants are less negatively affected by flooding when growing in species-rich plant communities. New Phytologist 213: 645–656. 27717024 10.1111/nph.14185

[CIT0080] Wright IJ , Reich PB, Westoby M, et al 2004. The worldwide leaf economics spectrum. Nature 428: 821–827. 15103368 10.1038/nature02403

