# Supplementary material for: Leading trait dimensions in flood-tolerant plants
Source: Ann Bot. 2022 Mar 8;130(3):383–92. doi: 10.1093/aob/mcac031 (PMC9486907; doi:10.1093/aob/mcac031)
Supplement: mcac031_suppl_Supplementary_Appendix_S1 [file mcac031_suppl_supplementary_appendix_s1.docx]

**The leading trait dimensions in flood-tolerant plants**

Yingji Pan*, Ellen Cieraad, Jean Armstrong, William Armstrong, Beverley R. Clarkson, Ole Pedersen, Eric J. W. Visser, Laurentius A.C.J. Voesenek, Peter M. van Bodegom

**Appendix A**: Derive Ellenberg moisture indicator values for plant species in analysis

When evaluating species performances along a gradient from dry to wet conditions, the Ellenberg moisture indicator is a useful summary of the general adaptations of a species (Ellenberg, 1988). It effectively represents the synergy of species adaptations to the complex adverse wetland (or drought) conditions. The Ellenberg moisture indicator classification consists of 12 levels corresponding to species occurrences along a gradient from 1 (species occurring in very dry habitats) to 12 (strictly aquatic plant species) (Ellenberg, 1988). Wetland plants usually occupy the higher range from level 4 (Shipley *et al.*, 2017) up to level 12 containing obligate aquatic plants. Studies have shown that the Ellenberg moisture indicator can be linked to plant functional traits and soil variables (Bartholomeus *et al.*, 2008; Bartelheimer & Poschlod, 2016; Shipley *et al.*, 2017).

In this study, the Ellenberg moisture indicator was obtained from both the European mainland (Ellenberg, 1988) and the British vegetation descriptions (Hill *et al.*, 2000). Moreover, to make the Ellenberg moisture indicator applicable for a global analysis, we related the Ellenberg moisture indicator values with the USDA wetland plant classification as proposed by Lichvar et al. 2016 (http://wetland-plants.usace.army.mil/). This system principally categorizes 8092 plant species occurring in the United States of America into five wetness indicator categories. The categories include sequentially Obligate (OBL) species with 99% occurrence in wetlands, Facultative Wetland (FACW) with 67%-99% occurrence in wetlands, Facultative (FAC) with 34%-66% occurrence in wetlands, Facultative Upland (FACU) with 1%-33% occurrence in wetlands, and Upland (UPL) with less than 1% occurrence in wetlands (Lichvar *et al.*, 2016). We coded the five USDA indicator categories from UPL to OBL into 1-5 ordinal classes and refer to this indicator system as the USDA indicator. All species selected for the analysis had an Ellenberg moisture or a USDA indicator value.

Using a simple linear regression of the Ellenberg moisture and the USDA indicator for the 328 plants common to both datasets, we were able to convert USDA indicators to Ellenberg values for all remaining species using the following relationship:

*Ellenberg moisture indicator= 1.6531*USDA indicator+1.5084 (R^2^=0.744, n=328)*

The combination of the original Ellenberg moisture values and the USDA indicators converted to Ellenberg values were used in the analysis described in the main text.

**References**

Bartelheimer, M. & Poschlod, P. (2016) Functional characterizations of Ellenberg indicator values - a review on ecophysiological determinants. *Functional Ecology*, **30**, 506–516.

Bartholomeus, R.P., Witte, J.-P.M., van Bodegom, P.M. & Aerts, R. (2008) The need of data harmonization to derive robust empirical relationships between soil conditions and vegetation. *Journal of Vegetation Science*, **19**, 799–808.

Ellenberg, H.H. (1988) *Vegetation ecology of central Europe*, 4th edn. Cambridge University Press, Cambridge.

Hill, M.O., Roy, D.B., Mountford, J.O. & Bunce, R.G.H. (2000) Extending Ellenberg’s indicator values to a new area: An algorithmic approach. *Journal of Applied Ecology*, **37**, 3–15.

Lichvar, R.W., Banks, D.L., Kirchner, W.N. & Melvin, N.C. (2016) The National Wetland Plant List: 2016 wetland ratings. *Phytoneuron*, **30**, 1–7.

Shipley, B., Belluau, M., Kühn, I., Soudzilovskaia, N.A., Bahn, M., Penuelas, J., Kattge, J., Sack, L., Cavender-Bares, J., Ozinga, W.A., Blonder, B., van Bodegom, P.M., Manning, P., Hickler, T., Sosinski, E., Pillar, V.D.P., Onipchenko, V. & Poschlod, P. (2017) Predicting habitat affinities of plant species using commonly measured functional traits. *Journal of Vegetation Science*, **28**, 1082–1095.
