# Supplementary material for: Leading trait dimensions in flood-tolerant plants
Source: Ann Bot. 2022 Mar 8;130(3):383–92. doi: 10.1093/aob/mcac031 (PMC9486907; doi:10.1093/aob/mcac031)
Supplement: mcac031_suppl_Supplementary_Appendix_S2 [file mcac031_suppl_supplementary_appendix_s2.docx]

| **The leading trait dimensions in flood-tolerant plants** | |  |  |  |  |  |  |  |  |
| --- | --- | --- | --- | --- | --- | --- | --- | --- | --- |
| Yingji Pan*, Ellen Cieraad, Jean Armstrong, William Armstrong, Beverley R. Clarkson, Ole Pedersen, Eric J. W. Visser, Laurentius A.C.J. Voesenek, Peter M. van Bodegom | | | | | |  |  |  |  |
|  |  |  |  |  |  |  |  |  |  |
| **Species name** | **Habitat type** | **Life form** | **Root porosity** | **Root/shoot ratio** | **Shoot elongation** | **leaf N** | **leaf P** | **SLA** | **Plant Height** |
| Acer campestre | temporary non-forested wetlands | shrub/tree | **×** |  |  | **×** | **×** | **×** | **×** |
| Acer negundo | forested/shrub wetlands | shrub/tree | **×** |  |  | **×** | **×** | **×** | **×** |
| Achillea millefolium | temporary non-forested wetlands | emergent | **×** | **×** | **×** | **×** | **×** | **×** | **×** |
| Acorus calamus | rivers and lakes | emergent | **×** | **×** | **×** | **×** | **×** | **×** | **×** |
| Aegopodium podagraria | forested/shrub wetlands | emergent | **×** |  |  | **×** | **×** | **×** | **×** |
| Agrostis capillaris | marsh | grass | **×** |  |  | **×** | **×** | **×** | **×** |
| Agrostis stolonifera | fen | grass | **×** | **×** | **×** | **×** | **×** | **×** | **×** |
| Ajuga reptans | forested/shrub wetlands | emergent | **×** |  |  | **×** | **×** | **×** | **×** |
| Alnus glutinosa | forested/shrub wetlands | shrub/tree | **×** |  |  | **×** | **×** | **×** | **×** |
| Alopecurus pratensis | forested/shrub wetlands | grass | **×** |  |  | **×** | **×** | **×** | **×** |
| Ammophila arenaria | forested/shrub wetlands | grass | **×** |  |  | **×** | **×** | **×** | **×** |
| Arrhenatherum elatius | temporary non-forested wetlands | grass |  | **×** | **×** | **×** | **×** | **×** | **×** |
| Avicennia germinans | temporary brackish/saline non-forested wetlands | shrub/tree | **×** | **×** |  | **×** | **×** | **×** | **×** |
| Avicennia marina | mangrove swamps | shrub/tree | **×** | **×** |  | **×** | **×** | **×** | **×** |
| Bidens cernua | marsh | emergent | **×** | **×** |  | **×** | **×** | **×** | **×** |
| Bidens frondosa | fen | emergent | **×** |  | **×** | **×** | **×** | **×** | **×** |
| Brachiaria mutica | temporary non-forested wetlands | grass | **×** | **×** |  | **×** | **×** | **×** | **×** |
| Calamagrostis canadensis | marsh | grass | **×** | **×** |  | **×** | **×** | **×** | **×** |
| Calamagrostis epigejos | temporary non-forested wetlands | grass | **×** | **×** |  | **×** | **×** | **×** | **×** |
| Calluna vulgaris | temporary non-forested wetlands | shrub/tree | **×** |  |  | **×** | **×** | **×** | **×** |
| Caltha palustris | fen | emergent | **×** |  | **×** | **×** | **×** | **×** | **×** |
| Calystegia sepium | forested/shrub wetlands | emergent | **×** |  |  | **×** | **×** | **×** | **×** |
| Carex acuta | marsh | sedge | **×** |  |  | **×** | **×** | **×** | **×** |
| Carex arenaria | forested/shrub wetlands | sedge | **×** | **×** |  | **×** | **×** | **×** | **×** |
| Carex crinita | temporary non-forested wetlands | sedge |  | **×** |  | **×** | **×** | **×** | **×** |
| Carex flacca | fen | sedge | **×** | **×** |  | **×** | **×** | **×** | **×** |
| Carex hirta | forested/shrub wetlands | sedge | **×** |  |  | **×** | **×** | **×** | **×** |
| Carex nigra | temporary non-forested wetlands | sedge | **×** | **×** |  | **×** | **×** | **×** | **×** |
| Carex remota | forested/shrub wetlands | sedge | **×** |  |  | **×** | **×** | **×** | **×** |
| Carex riparia | forested/shrub wetlands | sedge | **×** |  |  | **×** | **×** | **×** | **×** |
| Carex rostrata | temporary non-forested wetlands | sedge | **×** | **×** |  | **×** | **×** | **×** | **×** |
| Carpinus betulus | temporary non-forested wetlands | shrub/tree | **×** |  |  | **×** | **×** | **×** | **×** |
| Circaea lutetiana | forested/shrub wetlands | emergent | **×** |  |  | **×** | **×** | **×** | **×** |
| Cirsium arvense | forested/shrub wetlands | emergent | **×** | **×** | **×** | **×** | **×** | **×** | **×** |
| Cornus sanguinea | temporary non-forested wetlands | shrub/tree | **×** |  |  | **×** | **×** | **×** | **×** |
| Crataegus monogyna | forested/shrub wetlands | shrub/tree | **×** |  |  | **×** | **×** | **×** | **×** |
| Cyperus papyrus | marsh | sedge | **×** |  |  | **×** | **×** | **×** | **×** |
| Daucus carota | fen | emergent |  | **×** | **×** | **×** | **×** | **×** | **×** |
| Deschampsia flexuosa | forested/shrub wetlands | grass | **×** |  |  | **×** | **×** | **×** | **×** |
| Echinochloa crus-galli | temporary non-forested wetlands | grass | **×** | **×** | **×** | **×** | **×** | **×** | **×** |
| Elaeagnus rhamnoides | forested/shrub wetlands | shrub/tree | **×** |  |  | **×** | **×** | **×** | **×** |
| Eleocharis palustris | rivers and lakes | sedge | **×** | **×** |  | **×** | **×** | **×** | **×** |
| Elymus repens | forested/shrub wetlands | grass | **×** |  | **×** | **×** | **×** | **×** | **×** |
| Erica tetralix | temporary non-forested wetlands | shrub/tree | **×** |  |  | **×** | **×** | **×** | **×** |
| Eupatorium cannabinum | rivers and lakes | emergent | **×** |  |  | **×** | **×** | **×** | **×** |
| Eupatorium perfoliatum | temporary non-forested wetlands | emergent | **×** | **×** |  | **×** | **×** | **×** | **×** |
| Eutrochium maculatum | temporary non-forested wetlands | emergent | **×** | **×** |  | **×** | **×** | **×** | **×** |
| Festuca arundinacea | temporary non-forested wetlands | grass |  | **×** | **×** | **×** | **×** | **×** | **×** |
| Festuca rubra | marsh | grass | **×** |  | **×** | **×** | **×** | **×** | **×** |
| Filipendula ulmaria | fen | emergent | **×** |  |  | **×** | **×** | **×** | **×** |
| Frangula alnus | forested/shrub wetlands | shrub/tree | **×** |  |  | **×** | **×** | **×** | **×** |
| Fraxinus angustifolia | forested/shrub wetlands | shrub/tree | **×** |  |  | **×** | **×** | **×** | **×** |
| Galium mollugo | temporary non-forested wetlands | emergent | **×** |  |  | **×** | **×** | **×** | **×** |
| Galium odoratum | forested/shrub wetlands | emergent | **×** |  |  | **×** | **×** | **×** | **×** |
| Galium verum | fen | emergent | **×** |  |  | **×** | **×** | **×** | **×** |
| Glechoma hederacea | forested/shrub wetlands | emergent | **×** |  |  | **×** | **×** | **×** | **×** |
| Glyceria maxima | rivers and lakes | grass | **×** | **×** |  | **×** | **×** | **×** | **×** |
| Helianthus tuberosus | forested/shrub wetlands | emergent | **×** |  |  | **×** | **×** | **×** | **×** |
| Holcus lanatus | forested/shrub wetlands | grass | **×** | **×** | **×** | **×** | **×** | **×** | **×** |
| Hydrocotyle vulgaris | rivers and lakes | emergent | **×** |  |  | **×** | **×** | **×** | **×** |
| Juncus articulatus | fen | sedge | **×** | **×** |  | **×** | **×** | **×** | **×** |
| Juncus effusus | bog | sedge | **×** | **×** |  | **×** | **×** | **×** | **×** |
| Juncus subnodulosus | fen | sedge | **×** | **×** |  | **×** | **×** | **×** | **×** |
| Laguncularia racemosa | mangrove swamps | shrub/tree | **×** | **×** |  | **×** | **×** | **×** | **×** |
| Lamium galeobdolon | forested/shrub wetlands | emergent | **×** |  |  | **×** | **×** | **×** | **×** |
| Lamium maculatum | forested/shrub wetlands | emergent | **×** |  |  | **×** | **×** | **×** | **×** |
| Leersia oryzoides | marsh | grass |  | **×** |  | **×** | **×** | **×** | **×** |
| Lolium perenne | temporary non-forested wetlands | grass |  | **×** | **×** | **×** | **×** | **×** | **×** |
| Lotus corniculatus | fen | emergent | **×** |  |  | **×** | **×** | **×** | **×** |
| Lotus pedunculatus | rivers and lakes | emergent | **×** |  |  | **×** | **×** | **×** | **×** |
| Lycopus americanus | temporary non-forested wetlands | emergent |  | **×** |  | **×** | **×** | **×** | **×** |
| Lycopus europaeus | rivers and lakes | emergent | **×** |  | **×** | **×** | **×** | **×** | **×** |
| Lysimachia vulgaris | fen | emergent | **×** |  |  | **×** | **×** | **×** | **×** |
| Lythrum salicaria | rivers and lakes | emergent | **×** | **×** | **×** | **×** | **×** | **×** | **×** |
| Melampyrum sylvaticum | marsh | emergent | **×** |  |  | **×** | **×** | **×** | **×** |
| Mentha aquatica | fen | emergent | **×** | **×** |  | **×** | **×** | **×** | **×** |
| Mentha longifolia | forested/shrub wetlands | emergent | **×** |  |  | **×** | **×** | **×** | **×** |
| Molinia caerulea | fen | grass | **×** |  |  | **×** | **×** | **×** | **×** |
| Myriophyllum spicatum | rivers and lakes | submerged | **×** |  |  | **×** | **×** | **×** | **×** |
| Nardus stricta | temporary non-forested wetlands | grass | **×** | **×** |  | **×** | **×** | **×** | **×** |
| Nymphoides peltata | fen | floating-leaved | **×** | **×** | **×** | **×** | **×** | **×** | **×** |
| Persicaria amphibia | rivers and lakes | floating-leaved |  | **×** | **×** | **×** | **×** | **×** | **×** |
| Persicaria hydropiper | temporary non-forested wetlands | emergent |  | **×** | **×** | **×** | **×** | **×** | **×** |
| Persicaria lapathifolia | temporary non-forested wetlands | emergent |  | **×** | **×** | **×** | **×** | **×** | **×** |
| Phalaris arundinacea | forested/shrub wetlands | grass | **×** | **×** | **×** | **×** | **×** | **×** | **×** |
| Phleum pratense | forested/shrub wetlands | grass |  | **×** |  | **×** | **×** | **×** | **×** |
| Phormium tenax | marsh | grass | **×** | **×** |  | **×** | **×** | **×** | **×** |
| Phragmites australis | rivers and lakes | grass | **×** | **×** | **×** | **×** | **×** | **×** | **×** |
| Plantago lanceolata | temporary non-forested wetlands | emergent | **×** | **×** | **×** | **×** | **×** | **×** | **×** |
| Plantago major | rivers and lakes | emergent |  | **×** | **×** | **×** | **×** | **×** | **×** |
| Poa trivialis | forested/shrub wetlands | grass |  |  | **×** | **×** | **×** | **×** | **×** |
| Populus nigra | temporary non-forested wetlands | shrub/tree | **×** |  |  | **×** | **×** | **×** | **×** |
| Populus tremula | marsh | shrub/tree | **×** |  |  | **×** | **×** | **×** | **×** |
| Potentilla anserina | temporary non-forested wetlands | emergent | **×** | **×** |  | **×** | **×** | **×** | **×** |
| Potentilla reptans | forested/shrub wetlands | emergent | **×** |  | **×** | **×** | **×** | **×** | **×** |
| Prunus spinosa | marsh | shrub/tree | **×** |  |  | **×** | **×** | **×** | **×** |
| Quercus robur | temporary non-forested wetlands | shrub/tree | **×** |  |  | **×** | **×** | **×** | **×** |
| Ranunculus acris | marsh | emergent | **×** | **×** | **×** | **×** | **×** | **×** | **×** |
| Ranunculus repens | rivers and lakes | emergent |  |  | **×** | **×** | **×** | **×** | **×** |
| Reynoutria japonica | forested/shrub wetlands | emergent | **×** |  |  | **×** | **×** | **×** | **×** |
| Rhizophora mangle | mangrove swamps | shrub/tree | **×** | **×** |  | **×** | **×** | **×** | **×** |
| Rubus caesius | forested/shrub wetlands | emergent | **×** |  |  | **×** | **×** | **×** | **×** |
| Rumex acetosa | marsh | emergent | **×** | **×** | **×** | **×** | **×** | **×** | **×** |
| Rumex acetosella | temporary non-forested wetlands | emergent | **×** |  |  | **×** | **×** | **×** | **×** |
| Rumex crispus | marsh | emergent | **×** | **×** | **×** | **×** | **×** | **×** | **×** |
| Rumex verticillatus | marsh | emergent |  | **×** |  | **×** | **×** | **×** | **×** |
| Salix × fragilis | forested/shrub wetlands | shrub/tree | **×** |  |  | **×** | **×** | **×** | **×** |
| Salix caprea | marsh | shrub/tree | **×** |  |  | **×** | **×** | **×** | **×** |
| Salix cinerea | forested/shrub wetlands | shrub/tree | **×** |  |  | **×** | **×** | **×** | **×** |
| Salix purpurea | forested/shrub wetlands | shrub/tree | **×** |  |  | **×** | **×** | **×** | **×** |
| Salix repens | forested/shrub wetlands | shrub/tree | **×** |  |  | **×** | **×** | **×** | **×** |
| Salix triandra | forested/shrub wetlands | shrub/tree | **×** |  |  | **×** | **×** | **×** | **×** |
| Sambucus nigra | forested/shrub wetlands | shrub/tree | **×** |  |  | **×** | **×** | **×** | **×** |
| Schoenoplectus americanus | rivers and lakes | sedge |  | **×** |  | **×** | **×** | **×** | **×** |
| Schoenoplectus tabernaemontani | rivers and lakes | sedge | **×** | **×** |  | **×** | **×** | **×** | **×** |
| Scirpus cyperinus | marsh | sedge |  | **×** |  | **×** | **×** | **×** | **×** |
| Solanum dulcamara | rivers and lakes | emergent | **×** |  |  | **×** | **×** | **×** | **×** |
| Solidago gigantea | temporary brackish/saline non-forested wetlands | emergent | **×** | **×** |  | **×** | **×** | **×** | **×** |
| Sorbus aucuparia | forested/shrub wetlands | shrub/tree | **×** |  |  | **×** | **×** | **×** | **×** |
| Spartina alterniflora | temporary brackish/saline non-forested wetlands | grass | **×** | **×** |  | **×** | **×** | **×** | **×** |
| Spartina pectinata | temporary non-forested wetlands | grass | **×** | **×** |  | **×** | **×** | **×** | **×** |
| Succisa pratensis | temporary non-forested wetlands | emergent | **×** |  |  | **×** | **×** | **×** | **×** |
| Tanacetum vulgare | marsh | emergent | **×** |  |  | **×** | **×** | **×** | **×** |
| Trifolium repens | forested/shrub wetlands | emergent | **×** |  | **×** | **×** | **×** | **×** | **×** |
| Typha angustifolia | rivers and lakes | emergent | **×** | **×** |  | **×** | **×** | **×** | **×** |
| Typha domingensis | marsh | emergent | **×** |  |  | **×** | **×** | **×** | **×** |
| Typha latifolia | rivers and lakes | emergent | **×** | **×** |  | **×** | **×** | **×** | **×** |
| Ulmus minor | temporary non-forested wetlands | shrub/tree | **×** |  |  | **×** | **×** | **×** | **×** |
| Urtica dioica | forested/shrub wetlands | emergent | **×** |  |  | **×** | **×** | **×** | **×** |
| Verbena hastata | temporary non-forested wetlands | emergent |  | **×** |  | **×** | **×** | **×** | **×** |
| Xanthium strumarium | temporary non-forested wetlands | emergent | **×** | **×** | **×** | **×** | **×** | **×** | **×** |
|  |  |  |  |  |  |  |  |  |  |
| Note: "**×**" indicates record available |  |  |  |  |  |  |  |  |  |
|  |  |  |  |  |  |  |  |  |  |
| **Data sources** |  |  |  |  |  |  |  |  |  |
| Alongi, D.M., Clough, B.F. & Robertson, A.I. (2005) Nutrient-use efficiency in arid-zone forests of the mangroves Rhizophora stylosa and Avicennia marina. Aquatic Botany, 82, 121–131. | | | | | | | |  |  |
| Amber, unpublished |  |  |  |  |  |  |  |  |  |
| Armstrong, J. & Armstrong, W. (1991) A convective through-flow of gases in Phragmites australis (Cav.) Trin. ex Steud. Aquatic Botany, 39, 75–88. | | | | |  |  |  |  |  |
| Armstrong, J., Afreen-Zobayed, F., Blyth, S. & Armstrong, W. (1999) Phragmites australis: effects of shoot submergence on seedling growth and survival and radial oxygen loss from roots. Aquatic Botany, 64, 275–289. | | | | | | | | | |
| Armstrong, J., Armstrong, W. & Beckett, P.M. (1992) Phragmites australis: Venturi‐ and humidity‐induced pressure flows enhance rhizome aeration and rhizosphere oxidation. New Phytologist, 120, 197–207. | | | | | | | | | |
| Auclair, A.N.D. (1977) Factors affecting tissue nutrient concentrations in aCarex meadow. Oecologia, 28, 233–246. | | |  |  |  |  |  |  |  |
| Bakker, C., Rodenburg, J. & Van Bodegom, P.M. (2005) Effects of Ca- and Fe-rich seepage on P availability and plant performance in calcareous dune soils. Plant and Soil, 275, 111–122. | | | | | | | |  |  |
| Bakker, C., Van Bodegom, P.M., Nelissen, H.J.M., Ernst, W.H.O. & Aerts, R. (2006) Plant responses to rising water tables and nutrient management in calcareous dune slacks. Plant Ecology, 185, 19–28. | | | | | | | | |  |
| Baruch, Z. (1994) Responses to drought and flooding in tropical forage grasses; I. Biomass allocation, leaf growth and mineral nutrients. Plant and Soil, 164, 87–96. | | | | | |  |  |  |  |
| Baruch, Z., Mérida, T. & Merida, B.&. (1995) Effects of drought and flooding on root anatomy in four tropical forage grasses. International Journal of Plant Sciences, 156, 514–521. | | | | | | |  |  |  |
| Belluau, M., & Shipley, B. (2018). Linking hard and soft traits: Physiology, morphology and anatomy interact to determine habitat affinities to soil water availability in herbaceous dicots. Plos One, 13(3), e0193130. doi:10.1371/journal.pone.0193130 | | | | | | | | | |
| Blom, C., Voesenek, L.A.C.J., Banga, M., Engelaar, W.M.H.G., Rijnders, J.H.G.M., Van de Steeg, H.M. & Visser, E.J.W. (1994) Physiological Ecology of Riverside Species: Adaptive Responses of Plants to Submergence. Annals of Botany, 74, 253–263. | | | | | | | | | |
| Bowdish, T.I. & Stiling, P. (1998) The influence of salt and nitrogen on herbivore abundance: Direct and indirect effects. Oecologia, 113, 400–405. | | | | |  |  |  |  |  |
| Brian Atwell database. |  |  |  |  |  |  |  |  |  |
| Brix, H., Sorrell, B.K. & Orr, P.T. (1992) Internal pressurization and convective gas flow in some emergent freshwater macrophytes. Limnology and Oceanography, 37, 1420–1433. | | | | | | |  |  |  |
| Caines, L.A. (1965) The phosphorus content of some aquatic macrophytes with special reference to seasonal fluctuations and applications of phosphate fertilizers. Hydrobiologia, 25, 289–301. | | | | | | | |  |  |
| Cao, T., Ni, L., Xie, P., Xu, J., & Zhang, M. (2011). Effects of moderate ammonium enrichment on three submersed macrophytes under contrasting light availability. Freshwater Biology, 56(8), 1620–1629. doi:10.1111/j.1365-2427.2011.02601.x | | | | | | | | | |
| Cardona-Olarte, P., Twilley, R.R., Krauss, K.W. & Rivera-Monroy, V. (2006) Responses of neotropical mangrove seedlings grown in monoculture and mixed culture under treatments of hydroperiod and salinity. Hydrobiologia, 569, 325–341. | | | | | | | | | |
| Carpenter and Rejmankova, unpublished | |  |  |  |  |  |  |  |  |
| Chabbi, A., Mckee, K.L. & Mendelssohn, I.A. (2000) Fate of oxygen losses from Typha domingensis (Typhaceae) and Cladium jamaicense (Cyperaceae) and consequences for root metabolism. American Journal of Botany, 87, 1081–1090. | | | | | | | | | |
| Chapin III, F.S., Bret-Harte, M.S., Hobbie, S.E. & Zhong, H. (1996) Plant functional types as predictors of transient responses of arctic vegetation to global change. Journal of Vegetation Science, 7, 347–358. | | | | | | | | |  |
| Cheng, X., Peng, R., Chen, J., Luo, Y., Zhang, Q., An, S., Chen, J. & Li, B. (2007) CH4 and N2O emissions from Spartina alterniflora and Phragmites australis in experimental mesocosms. Chemosphere, 68, 420–427. | | | | | | | | | |
| Clarkson, unpublished |  |  |  |  |  |  |  |  |  |
| Clarkson, unpublished update |  |  |  |  |  |  |  |  |  |
| Clough, B. (1982) Mangrove Ecosystems in Australia. Structure, Function and Management. Australian National University Press. | | | |  |  |  |  |  |  |
| Colmer, T.D. & Pedersen, O. (2008) Underwater photosynthesis and respiration in leaves of submerged wetland plants: gas films improve CO2 and O2 exchange. New Phytol, 177, 918–926. | | | | | | | |  |  |
| Curran, M. (1985) Gas movements in the roots of Avicennia marina (Forsk.) Vierh. Australian Journal of Plant Physiology, 12, 97–108. | | | | |  |  |  |  |  |
| Curtis, P. S., Drake, B. G., Leadley, P. W., Arp, W. J., & Whigham, D. F. (1989). Growth and senescence in plant communities exposed to elevated CO2 concentrations on an estuarine marsh. Oecologia, 78(1), 20–26. doi:10.1007/BF00377193 | | | | | | | | | |
| Ellison, A. M., & Farnsworth, E. J. (1996). Spatial and temporal variability in growth of Rhizophora Mangle saplings on Coral Cays: Links with variation in insolation, herbivory, and local sedimentation rate. The Journal of Ecology, 84(5), 717. https://doi.org/10.2307/2261334 | | | | | | | | | |
| Ellison, A.M. & Farnsworth, E.J. (1997) Simulated sea level change alters anatomy, physiology, growth, and reproduction of red mangrove ( Rhizophora mangle L.). Oecologia, 112, 435–446. | | | | | | | |  |  |
| Ellison, A.M. (2002) Macroecology of mangroves: large-scale patterns and processes in tropical coastal forests. Trees, 16, 181–194. | | | | |  |  |  |  |  |
| Fajer, E.D., Bowers, M.D. & Bazzaz, F.A. (1989) The effects of enriched carbon dioxide atmospheres on plant--Insect herbivore interactions. Science, 243, 1198–1200. | | | | | |  |  |  |  |
| Farnsworth, E.J. & Meyerson, L.A. (2003) Comparative ecophysiology of four wetland plant species along a continuum of invasiveness. Wetlands, 23, 750–762. | | | | | |  |  |  |  |
| Farnsworth, E.J., Ellison, A.M. & Gong, W.K. (1996) Elevated CO2 alters anatomy, physiology, growth, and reproduction of red mangrove (Rhizophora mangle L.). Oecologia, 108, 599–609. | | | | | | | |  |  |
| from Rejmankova data compilation |  |  |  |  |  |  |  |  |  |
| Frye, J. & Grosse, W. (1992) Growth-Responses To Flooding and Recovery of Deciduous Trees. Zeitschrift Fur Naturforschung C-a Journal of Biosciences, 47, 683–689. | | | | | |  |  |  |  |
| Fu, H., Yuan, G., Cao, T., Ni, L., Li, W., & Zhu, G. (2012). Relationships between relative growth rate and its components across 11 submersed macrophytes. Journal of Freshwater Ecology, 27(4), 471–480. doi:10.1080/02705060.2012.684102 | | | | | | | | | |
| Fu, H., Zhong, J., Yuan, G., Xie, P., Guo, L., Zhang, X., … Ni, L. (2014). Trait-based community assembly of aquatic macrophytes along a water depth gradient in a freshwater lake. Freshwater Biology, 59(12), 2462–2471. doi:10.1111/fwb.12443 | | | | | | | | | |
| Güsewell, S. & Koerselman, W. (2002) Variation in nitrogen and phosphorus concentrations of wetland plants. Perspectives in Plant Ecology, Evolution and Systematics, 5, 37–61. | | | | | | |  |  |  |
| Gusewell, S., Bollens, U., Ryser, P. & Klotzli, F. (2003) Contrasting effects of nitrogen, phosphorus and water regime on first- and second-year growth of 16 wetland plant species. Functional Ecology, 17, 754–765. | | | | | | | | | |
| Güsewell, S., Koerselman, W. & Verhoeven, J.T.A. (2003) Biomass N:P ratios as indicators of nutrient limitation for plant populations in wetlands. Ecological Applications, 13, 372–384. | | | | | | |  |  |  |
| Güsewell, S., Zuberbühler, N. & Clerc, C. (2005) Distribution and functional traits of Solidago gigantea in a Swiss lakeshore wetland. Botanica Helvetica, 115, 63–75. | | | | | |  |  |  |  |
| Herzog, M. & Pedersen, O. (2014) Partial versus complete submergence: Snorkelling aids root aeration in Rumex palustris but not in R.acetosa. Plant, Cell and Environment, 37, 2381–2390. | | | | | | | |  |  |
| Huber, H., Jacobs, E., & Visser, E. J. W. (2009). Variation in flooding-induced morphological traits in natural populations of white clover (Trifolium repens) and their effects on plant performance during soil flooding. Annals of Botany, 103(2), 377–386. doi:10.1093/aob/mcn149 | | | | | | | | | |
| Johnston, A. & Bezeau, L.M. (1962) Chemical composition of range forage plants of the Festuca Scabrella association. Canadian Journal of Plant Science, 42, 105–115. | | | | | |  |  |  |  |
| Jones, M.B. (1988) Photosynthetic Responses of C 3 and C 4 Wetland Species in a Tropical Swamp. The Journal of Ecology, 76, 253–262. | | | | |  |  |  |  |  |
| Jung, V., Hoffmann, L., & Muller, S. (2009). Ecophysiological responses of nine floodplain meadow species to changing hydrological conditions. Plant Ecology, 201(2), 589–598. doi:10.1007/s11258-008-9508-9 | | | | | | | | | |
| Justin, S.H.F.W. & Armstrong, W. (1987) The anatomical characteristics of roots and plant response to soil flooding. New Phytologist, 106, 465–495. | | | | |  |  |  |  |  |
| Keddy, P., Fraser, L.H. & Wisheu, I.C. (1998) A comparative approach to examine competitive response of 48 wetland plant species. Journal of Vegetation Science, 9, 777–786. | | | | | | |  |  |  |
| Kercher, S.M. & Zedler, J.B. (2004) Flood tolerance in wetland angiosperms: a comparison of invasive and noninvasive species. Aquatic Botany, 80, 89–102. | | | | | |  |  |  |  |
| Konnerup, D., Sorrell, B. K., & Brix, H. (2011). Do tropical wetland plants possess convective gas flow mechanisms? New Phytologist, 190(2), 379–386. doi:10.1111/j.1469-8137.2010.03585.x | | | | | | | |  |  |
| Kotowski, W., Beauchard, O., Opdekamp, W., Meire, P., & Van Diggelen, R. (2010). Waterlogging and canopy interact to control species recruitment in floodplains. Functional Ecology, 24(4), 918–926. doi:10.1111/j.1365-2435.2009.01682.x | | | | | | | | | |
| Krauss, K.W., Twilley, R.R., Doyle, T.W. & Gardiner, E.S. (2006) Leaf gas exchange characteristics of three neotropical mangrove species in response to varying hydroperiod. Tree Physiology, 26, 959–968. | | | | | | | | |  |
| Laan, P., Berrevoets, M.J., Lythe, S., Armstrong, W. & Blom, C.W.P.M. (1989) Root Morphology and Aerenchyma Formation as Indicators of the Flood-Tolerance of Rumex Species. The Journal of Ecology, 77, 693–703. | | | | | | | | | |
| Lai, W.-L., Wang, S.-Q., Peng, C.-L., & Chen, Z.-H. (2011). Root features related to plant growth and nutrient removal of 35 wetland plants. Water Research, 45(13), 3941–3950. doi:10.1016/j.watres.2011.05.002 | | | | | | | | | |
| Lemoine, D. G., Mermillod-Blondin, F., Barrat-Segretain, M.-H., Massé, C., & Malet, E. (2012). The ability of aquatic macrophytes to increase root porosity and radial oxygen loss determines their resistance to sediment anoxia. Aquatic Ecology, 46(2), 191–200. doi:10.1007/s10452-012-9391-2 | | | | | | | | | |
| Lenssen, J.P.M., Menting, F.B.J., Van der Putten, W.H. & Blom, C.W.P.M. (2000) Vegetative reproduction by species with different adaptations to shallow-flooded habitats. New Phytologist, 145, 61–70. | | | | | | | | |  |
| Lenssen, J.P.M., ten Dolle, G.E. & Blom, C.W.P.M. (1998) The effect of flooding on the recruitment of reed marsh and tall forb plant species. Plant Ecology, 139, 13–23. | | | | | |  |  |  |  |
| Li, S.N., Cui, L.J., Song, H.T., Zhang, Y., Gao, C.J., Guo, J., Wei, W., Zhao, X.S., Zhang, M.Y., Wang, Y.F., and Li, W., 2012. Comparison on purification capacity of soil nitrogen and phosphorus in different wetland plants. Ecology and Environmental Sciences, 21(11);; 1870-1874 | | | | | | | | | |
| Li, W., Cao, T., Ni, L., Zhang, X., Zhu, G., & Xie, P. (2013). Effects of water depth on carbon, nitrogen and phosphorus stoichiometry of five submersed macrophytes in an in situ experiment. Ecological Engineering, 61, 358–365. doi:10.1016/j.ecoleng.2013.09.028 | | | | | | | | | |
| Li, Z., Xu, J., Cao, T., Ni, L., & Xie, P. (2010). Adaptive responses of a floating-leaved macrophyte,Nymphoides peltata, to a terrestrial habitat. Journal of Freshwater Ecology, 25(3), 481–486. doi:10.1080/02705060.2010.9664392 | | | | | | | | | |
| Li, Z., Yu, D., & Xu, J. (2010). Adaptation to water level variation: Responses of a floating-leaved macrophyteNymphoides peltatato terrestrial habitats. Annales de Limnologie - International Journal of Limnology, 47(1), 97–102. doi:10.1051/limn/2010029 | | | | | | | | | |
| Lovelock, C.E., Feller, I.C., Ball, M.C., Engelbrecht, B.M. & Ewe, M.L. (2006) Differences in plant function in phosphorus- and nitrogen-limited mangrove ecosystems. New Phytol, 172, 514–522. | | | | | | | |  |  |
| Lu, J., Zhou, H.X., Tian, G.Y. and Liu, G.H., 2011. Nitrogen and phosphorus contents in 44 wetland species from the Lake Erhai Basin. Acta Ecologica Sinica, 31(3):709-715 | | | | | |  |  |  |  |
| Luo FL, Huang L, Lei T, Xue W, Li HL, Yu FH, Cornelissen JHC. 2016. Responsiveness of performance and morphological traits to experimental submergence predicts field distribution pattern of wetland plants. Journal of Vegetation Science 27: 340–351. | | | | | | | | | |
| Mantlana, K.B., Arneth, A., Veenendaal, E.M., Wohland, P., Wolski, P., Kolle, O., Wagner, M. & Lloyd, J. (2008) Photosynthetic properties of C4 plants growing in an African savanna/wetland mosaic. J Exp Bot, 59, 3941–3952. | | | | | | | | | |
| Mason, C.F. & Bryant, R.J. (1975) Production, nutrient content and decomposition of Phragmites Communis Trin. and Typha Angustifolia L. The Journal of Ecology, 63, 71. | | | | | |  |  |  |  |
| McDonald, M.P., Galwey, N.W. & Colmer, T.D. (2002) Similarity and diversity in adventitious root anatomy as related to root aeration among a range of wetland and dryland grass species. Plant, Cell and Environment, 25, 441–451. | | | | | | | | | |
| McJannet, C.L., Keddy, P.A. & Pick, F.R. (1995) Nitrogen and Phosphorus Tissue Concentrations in 41 Wetland Plants: A Comparison Across Habitats and Functional Groups. Functional Ecology, 9, 231–238. | | | | | | | | |  |
| McKee, K.L. (1993) Soil physicochemical patterns and mangrove species distribution-reciprocal effects? The Journal of Ecology, 81, 477–487. | | | | |  |  |  |  |  |
| Mckee, K.L., Mendelssohn, I.A. & Burdick, D.M. (1989) Effect of long-term flooding on root metabolic response in five freshwater marsh plant species. Canadian Journal of Botany, 67, 3446–3452. | | | | | | | | |  |
| Medina, E., & Francisco, M. (1997). Osmalility and S13C of Leaf Tissues of Mangrove Species from Environments of Contrasting Rainfalland Salinity. Estuarine, Coastal and Shelf Science, 45, 337–344. | | | | | | | | |  |
| Mei, X.-Q., Yang, Y., Tam, N. F.-Y., Wang, Y.-W., & Li, L. (2014). Roles of root porosity, radial oxygen loss, Fe plaque formation on nutrient removal and tolerance of wetland plants to domestic wastewater. Water Research, 50, 147–159. doi:10.1016/j.watres.2013.12.004 | | | | | | | | | |
| Mendelssohn, I.A., McKee, K.L. & Patrick, W.H. (1981) Oxygen deficiency in Spartina alterniflora roots: metabolic adaptation to anoxia. Science, 214, 439–441. | | | | | |  |  |  |  |
| Méndez-Alonzo, R., López-Portillo, J. & Rivera-Monroy, V.H. (2008) Latitudinal Variation in Leaf and Tree Traits of the Mangrove Avicennia germinans (Avicenniaceae) in the Central Region of the Gulf of Mexico. Biotropica, 40, 449–456. | | | | | | | | | |
| Mozdzer, T. J., & Zieman, J. C. (2010). Ecophysiological differences between genetic lineages facilitate the invasion of non-nativePhragmites australisin North American Atlantic coast wetlands. Journal of Ecology, 98(2), 451–458. doi:10.1111/j.1365-2745.2009.01625.x | | | | | | | | | |
| Naidoo, G., McKee, K.L. & Mendelssohn, I.A. (1992) Anatomical and metabolic responses to waterlogging and salinity in Spartina alterniflora and S. patens (Poaceae). American Journal of Botany, 79, 765–770. | | | | | | | | | |
| Naidoo, G., Tuffers, A. V. & Von Willert, D.J. (2001) Changes in gas exchange and chlorophyll fluorescence characteristics of two mangroves and a mangrove associate in response to salinity in the natural environment. Trees, 16, 140–146. | | | | | | | | | |
| Nicol & Ganf Unpublished |  |  |  |  |  |  |  |  |  |
| Nielsen, S.L. & Sand-Jensen, K. (1989) Regulation of photosynthetic rates of submerged rooted macrophytes. Oecologia, 81, 364–368. | | | | |  |  |  |  |  |
| Nielsen, S.L. (1993) A comparison of aerial and submerged photosynthesis in some Danish amphibious plants. Aquatic Botany, 45, 27–40. | | | | |  |  |  |  |  |
| Ordoñez, J.C., Van Bodegom, P.M., Witte, J.-P.M., Bartholomeus, R.P., Van Dobben, H.F. & Aerts, R. (2010) Leaf habit and woodiness regulate different leaf economy traits at a given nutrient supply. Ecology, 91, 3218–3228. | | | | | | | | | |
| Pedersen unpublished data |  |  |  |  |  |  |  |  |  |
| Pezeshki, S.R., DeLaune, R.D. & Meeder, J.F. (1997) Carbon assimilation and biomass partitioning in Avicennia germinans and Rhizophora mangle seedlings in response to soil redox conditions. Environmental and Experimental Botany, 37, 161–171. | | | | | | | | | |
| Pi, N., Tam, N.F.Y., Wu, Y. & Wong, M.H. (2009) Root anatomy and spatial pattern of radial oxygen loss of eight true mangrove species. Aquatic Botany, 90, 222–230. | | | | | |  |  |  |  |
| Pierce, S., Brusa, G., Sartori, M., & Cerabolini, B. E. (2012). Combined use of leaf size and economics traits allows direct comparison of hydrophyte and terrestrial herbaceous adaptive strategies. Annals of Botany, 109(5), 1047–1053. doi:10.1093/aob/mcs021 | | | | | | | | | |
| Pierik unpublished |  |  |  |  |  |  |  |  |  |
| Poorter, H. & Remkes, C. (1990) Leaf area ratio and net assimilation rate of 24 wild species differing in relative growth rate. Oecologia, 83, 553–559. | | | | |  |  |  |  |  |
| Quested, H.M., Cornelissen, J.H.C., Press, M.C., Callaghan, T. V., Aerts, R., Trosien, F., Riemann, P., Gwynn-Jones, D., Kondratchuk, A. & Jonasson, S.E. (2003) Decomposition of sub-arctic plants with differing nitrogen economies: A functional role for hemiparasites. Ecology, 84, 3209–3221. | | | | | | | | | |
| Rao, R. G., Woitchik, A. F., Goeyens, L., Vanriet, A., Kazungu, J., & Dehairs, F. (1994). Carbon, Nitrogen Contents and Stable Carbon-Isotope Abundance in Mangrove Leaves From an East-African Coastal Lagoon (Kenya). Aquatic Botany, 47(2), 175–183. https://doi.org/10.1016/0304-3770(94)90012-4 | | | | | | | | | |
| Rejmankova, Eliska database I. |  |  |  |  |  |  |  |  |  |
| Rejmankova, Eliska database II. |  |  |  |  |  |  |  |  |  |
| Rejmankova, unpublished |  |  |  |  |  |  |  |  |  |
| Rejmankova-Okavango database, unpublished | |  |  |  |  |  |  |  |  |
| Ridge, I. (1987) Ethylene and growth control in amphibious plants. Plant life in aquatic and amphibious habitats (ed R.M.M. Crawford), pp. 53–76. British Ecological Society. | | | | | |  |  |  |  |
| Ronzhina, D. A., & Ivanov, L. A. (2014). Construction costs and mesostructure of leaves in hydrophytes. Russian Journal of Plant Physiology, 61(6), 776–783. doi:10.1134/s102144371406017x | | | | | | | |  |  |
| root porosity douma unpublished |  |  |  |  |  |  |  |  |  |
| Sand-Jensen K, Pedersen MF, Nielsen SL. 1992. Photosynthetic use of inorganic carbon among primary and secondary water plants in streams. Freshwater Biology 27: 283–293. | | | | | | |  |  |  |
| Sher-Kaul, S., Oertli, B., Castella, E., & Lachavanne, J.-B. (1995). Relationship between biomass and surface area of six submerged aquatic plant species. Aquatic Botany, 51(1–2), 147–154. doi:10.1016/0304-3770(95)00460-H | | | | | | | | | |
| Shipley, B. & Lechowicz, M.J. (2000) The functional co-ordination of leaf morphology, nitrogen concentration, and gas exchange in40 wetland species. Écoscience, 7, 183–194. | | | | | | |  |  |  |
| Shipley, B. & Peters, R.H. (1990) A Test of the Tilman Model of Plant Strategies: Relative Growth Rate and Biomass Partitioning. The American Naturalist, 136, 139–153. | | | | | |  |  |  |  |
| Shipley, B. & Vu, T.-T. (2002) Dry matter content as a measure of dry matter concentration in plants and their parts. New Phytologist, 153, 359–364. | | | | |  |  |  |  |  |
| Shipley, B., Keddy, P.A., Moore, D.R.J. & Lemky, K. (1989) Regeneration and Establishment Strategies of Emergent Macrophytes. The Journal of Ecology, 77, 1093–1110. | | | | | |  |  |  |  |
| Siebentritt & Ganf unpublished |  |  |  |  |  |  |  |  |  |
| Small, E. (1972) Ecological significance of four critical elements in plants of raised Spagnum peat bogs. Ecology, 53, 498–503. | | | |  |  |  |  |  |  |
| Smirnoff, N. & Crawford, R.M.M. (1983) Variation in the Structure and Response to Flooding of Root Aerenchyma in some Wetland Plants. Annals of Botany, 51, 237–249. | | | | | |  |  |  |  |
| Smith, J. A. C., Popp, M., Luttge, U., Cram, W. J., Diaz, M., Griffiths, H., … Thonke, B. (1989). Ecophysiology of xerophyte and halophyte vegetation of a coastal alluvial plain in northern Venezuela VI. Water relations and gas exchange of mangroves. New Phytologist, 111, 293–307. https://doi.org/10.1111/j.1469-8137.1989.tb00693.x | | | | | | | | | |
| Smits, A.J.M., Laan, P., Thier, R.H. & van der Velde, G. (1990) Root aerenchyma, oxygen leakage patterns and alcoholic fermentation ability of the roots of some nymphaeid and isoetid macrophytes in relation to the sediment type of their habitat. Aquatic Botany, 38, 3–17. | | | | | | | | | |
| Sobrado, M.A. (2005) Leaf characteristics and gas exchange of the mangrove Laguncularia racemosa as affected by salinity. Photosynthetica, 43, 217–221. | | | | | |  |  |  |  |
| Sorrell & Brix unpublished data |  |  |  |  |  |  |  |  |  |
| Sorrell & McKee unpublished data |  |  |  |  |  |  |  |  |  |
| Sorrell unpublished |  |  |  |  |  |  |  |  |  |
| Sorrell, B., Mendelssohn, I. A., Mckee, K. L., & Woods, R. A. (2000). Ecophysiology of wetland plant roots: A modelling comparison of aeration in relation to species distribution. Annals of Botany, 86(3), 675–685. doi:10.1006/anbo.2000.1173 | | | | | | | | | |
| Striker, G.G., Insausti, P., Grimoldi, A.A. & Vega, A.S. (2007) Trade-off between root porosity and mechanical strength in species with different types of aerenchyma. Plant, Cell & Environment, 30, 580–589. | | | | | | | | |  |
| Suárez, N. & Medina, E. (2006) Influence of salinity on Na+ and K+ accumulation, and gas exchange in Avicennia germinans. Photosynthetica, 44, 268–274. | | | | | |  |  |  |  |
| Sutton-Grier, A. E., & Megonigal, J. P. (2011). Plant species traits regulate methane production in freshwater wetland soils. Soil Biology and Biochemistry, 43(2), 413–420. doi:10.1016/j.soilbio.2010.11.009 | | | | | | | | |  |
| Taub, D.R. (2002) Analysis of interspecific variation in plant growth responses to nitrogen. Canadian Journal of Botany, 80, 34–41. | | | |  |  |  |  |  |  |
| Teakle, N.L., Real, D. & Colmer, T.D. (2006) Growth and ion relations in response to combined salinity and waterlogging in the perennial forage legumes Lotus corniculatus and Lotus tenuis. Plant and Soil, 289, 369–383. | | | | | | | | | |
| Thomas, A.T. & Hodkinson, I.D. (1991) Nitrogen, water stress and the feeding efficiency of lepidopteran herbivores. The Journal of Applied Ecology, 28, 703. | | | | | |  |  |  |  |
| Thomas, S.C. & Bazzaz, F.A. (1996) Elevated CO2 and leaf shape: Are dandelions getting toothier? American Journal of Botany, 83, 106–111. | | | | |  |  |  |  |  |
| Turner, I.M. & Tan, H.T.W. (1991) Habitat-related variation in tree leaf form in four tropical forest types on Pulau Ubin, Singapore. Journal of Vegetation Science, 2, 691–698. | | | | | | |  |  |  |
| Van Bodegom, P., Kanter, M. de, Bakker, C. & Aerts, R. (2005) Radial oxygen loss, a plastic property of dune slack plant species. Plant and Soil, 271, 351–364. | | | | | |  |  |  |  |
| Van Bodegom, P.M., Sorrell, B.K., Oosthoek, A., Bakker, C. & Aerts, R. (2008) Separating the effects of partial submergence and soil oxygen demand on plant physiology. Ecology, 89, 193–204. | | | | | | | |  |  |
| van Bodegom, unpublished |  |  |  |  |  |  |  |  |  |
| van Bodegom, unpublished Ukraine |  |  |  |  |  |  |  |  |  |
| Van Eck, W.H.J.M., Van de Steeg, H.M., Blom, C.W.P.M. & De Kroon, H. (2004) Is tolerance to summer flooding correlated with distribution patterns in river floodplains? A comparative study of 20 terrestrial grassland species. Oikos, 107, 393–405. | | | | | | | | | |
| Vincent, unpublished |  |  |  |  |  |  |  |  |  |
| Visser, E.J.W., Blom, C.W.P.M. & Voesenek, L.A.C.J. (1996) Flooding-induced adventitious rooting in Rumex: morphology and development in an ecological perspective. Acta Botanica Neerlandica, 45, 17–28. | | | | | | | | | |
| Visser, E.J.W., Bögemann, G.M., Van De Steeg, H.M., Pierik, R. & Blom, C.W.P.M. (2000) Flooding tolerance of Carex species in relation to field distribution and aerenchyma formation. New Phytologist, 148, 93–103. | | | | | | | | | |
| Visser, E.J.W., Colmer, T.D., Blom, C.W.P.M. & Voesenek, L.A.C.J. (2000) Changes in growth, porosity, and radial oxygen loss from adventitious roots of selected mono- and dicotyledonous wetland species with contrasting types of aerenchyma. Plant, Cell and Environment, 23, 1237–1245. | | | | | | | | | |
| Voesenek, L.A.C.J., Rijnders, J.H.G.M., Peeters, A.J.M., van de Steeg, H.M. & de Kroon, H. (2004) Plant hormones regulate fast shoot elongation under water: From genes to communities. Ecology, 85, 16–27. | | | | | | | | |  |
| Wang, Z., Xia, C., Yu, D., & Wu, Z. (2015). Low-temperature induced leaf elements accumulation in aquatic macrophytes across Tibetan Plateau. Ecological Engineering, 75, 1–8. doi:10.1016/j.ecoleng.2014.11.015 | | | | | | | | | |
| Weiher, unpublished |  |  |  |  |  |  |  |  |  |
| White, S.D. & Ganf, G.G. (1998) The influence of convective flow on rhizome length in Typha domingensis over a water depth gradient. Aquatic Botany, 62, 57–70. | | | | | |  |  |  |  |
| Winkel, A., Visser, E.J.W., Colmer, T.D., Brodersen, K.P., Voesenek, L.A.C.J., Sand-Jensen, K. & Pedersen, O. (2016) Leaf gas films, underwater photosynthesis and plant species distributions in a flood gradient. Plant, Cell and Environment, 39, 1537–1548. | | | | | | | | | |
| Wooller, M., Smallwood, B., Jacobson, M. & Fogel, M. (2003) Carbon and nitrogen stable isotopic variation in Laguncularia racemosa (L.) (white mangrove) from Florida and Belize: implications for trophic level studies. Hydrobiologia, 499, 13–23. | | | | | | | | | |
| Xing W, Wu H-P, Hao B-B, Liu G-H. 2013. Stoichiometric characteristics and responses of submerged macrophytes to eutrophication in lakes along the middle and lower reaches of the Yangtze River. Ecological Engineering 54: 16–21. | | | | | | | | | |
| Xiong, S., Nilsson, C., Johansson, M.E. & Jansson, R. (2001) Responses of riparian plants to accumulation of silt and plant litter: the importance of plant traits. Journal of Vegetation Science, 12, 481–490. | | | | | | | | |  |
| Yan, Z., Wang, W. & Tang, D. (2007) Effect of different time of salt stress on growth and some physiological processes of Avicennia marina seedlings. Marine Biology, 152, 581–587. | | | | | | |  |  |  |
| Youssef, T. & Saenger, P. (1996) Anatomical Adaptive Strategies to Flooding and Rhizosphere Oxidation in Mangrove Seedlings. Australian Journal of Botany, 44, 297–313. | | | | | | |  |  |  |
| Zhu, G., Li, W., Zhang, M., Ni, L., & Wang, S. (2012). Adaptation of submerged macrophytes to both water depth and flood intensity as revealed by their mechanical resistance. Hydrobiologia, 696(1), 77–93. doi:10.1007/s10750-012-1185-y | | | | | | | | | |
