# Supplementary material for: Leading trait dimensions in flood-tolerant plants
Source: Ann Bot. 2022 Mar 8;130(3):383–92. doi: 10.1093/aob/mcac031 (PMC9486907; doi:10.1093/aob/mcac031)
Supplement: mcac031_suppl_Supplementary_Appendix_S3 [file mcac031_suppl_supplementary_appendix_s3.docx]

**The leading trait dimensions in flood-tolerant plants**

**Appendix C**:


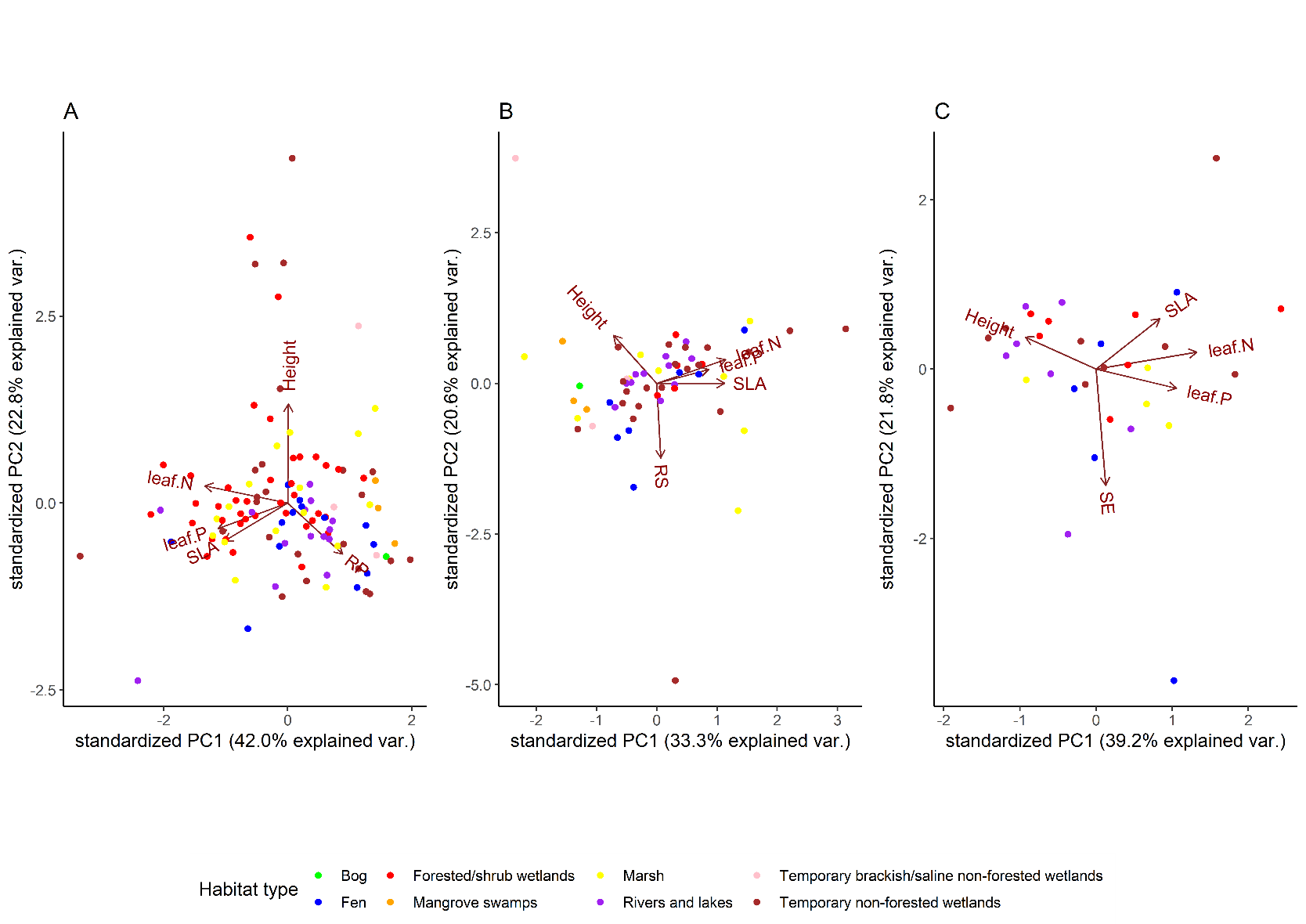


Figure S1 Principal Component Analysis (PCA) of leaf nitrogen, leaf phosphorus, specific leaf area, plant height and (A) root porosity, (B) root/shoot ratio and (C) shoot elongation labelled by habitat type.


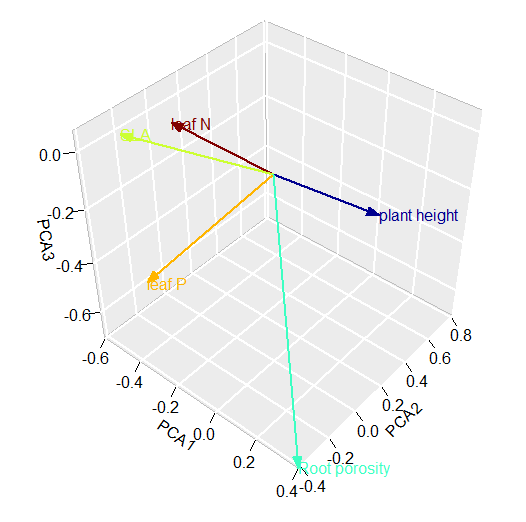

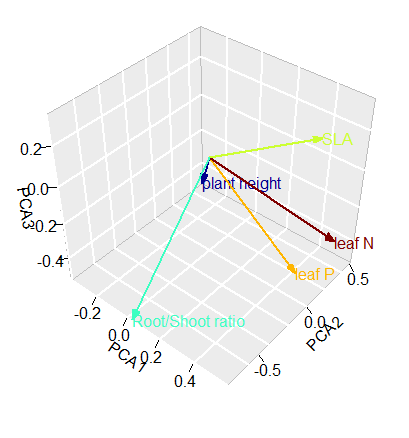

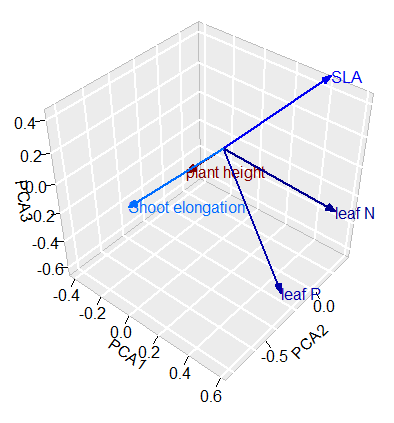


**（A）**

**（B）**

**（C）**

Figure S2 Three-dimensional Principal Component Analysis (PCA) plots of leaf nitrogen, leaf phosphorus, specific leaf area, plant height and (A) root porosity, (B) root/shoot ratio and (C) shoot elongation.

Table S1 Results of one-way ANOVA (F and P values) from three flooding-induced traits (root porosity, root/shoot ratio, and shoot elongation) effects on Ellenberg moisture indicator.

| Flooding-induced traits | df | F value | P value |
| --- | --- | --- | --- |
| Root porosity | 1 | 49.35 | <0.001 |
| Log_10_(Root/shoot ratio) | 1 | 0.105 | 0.75 |
| Log_10_(Shoot elongation) | 1 | 0.197 | 0.66 |

Note: Root/shoot ratio and shoot elongation are log-transformed before analysis to comply to a normal distribution and homogeneity of variance.
